# Genomic Predictions for Common Bunt, FHB, Stripe Rust, Leaf Rust, and Leaf Spotting Resistance in Spring Wheat

**DOI:** 10.3390/genes13040565

**Published:** 2022-03-23

**Authors:** Kassa Semagn, Muhammad Iqbal, Diego Jarquin, José Crossa, Reka Howard, Izabela Ciechanowska, Maria Antonia Henriquez, Harpinder Randhawa, Reem Aboukhaddour, Brent D. McCallum, Anita L. Brûlé-Babel, Alireza Navabi, Amidou N’Diaye, Curtis Pozniak, Dean Spaner

**Affiliations:** 1 Department of Agricultural, Food, and Nutritional Science, 4-10 Agriculture-Forestry Centre, University of Alberta, Edmonton, AB T6G 2P5, Canada; fentaye@ualberta.ca (K.S.); mi1@ualberta.ca (M.I.); izabela@ualberta.ca (I.C.); 2 Agronomy Department, University of Florida, Gainesville, FL 32611, USA; jhernandezjarqui@ufl.edu; 3 International Maize and Wheat Improvement Center (CIMMYT), Apdo. Postal 6-641, Mexico 06600, Mexico; j.crossa@cgiar.org; 4 Department of Statistics, University of Nebraska–Lincoln, Lincoln, NE 68583, USA; rekahoward@unl.edu; 5 Morden Research and Development Centre, Agriculture and Agri-Food Canada, 101 Route 100, Morden, MB R6M 1Y5, Canada; mariaantonia.henriquez@agr.gc.ca (M.A.H.); brent.mccallum@agr.gc.ca (B.D.M.); 6 Lethbridge Research and Development Centre, Agriculture and Agri-Food Canada, 5403-1st Avenue South, Lethbridge, AB T1J 4B1, Canada; harpinder.randhawa@agr.gc.ca (H.R.); reem.aboukhaddour@canada.ca (R.A.); 7 Department of Plant Science, University of Manitoba, 66 Dafoe Road, Winnipeg, MB R3T 2N2, Canada; anita.brule-babel@umanitoba.ca; 8 Department of Plant Agriculture, Crop Science Building, University of Guelph, Guelph, ON N1G 2W1, Canada; 9 Crop Development Centre and Department of Plant Sciences, University of Saskatchewan, 51 Campus Drive, Saskatoon, SK S7N 5A8, Canada; amidou.ndiaye@usask.ca (A.N.); curtis.pozniak@usask.ca (C.P.)

**Keywords:** 90K array, DArTseq, disease resistance, genomic selection, prairie provinces, prediction accuracy, priority wheat disease, SNP

## Abstract

Some studies have investigated the potential of genomic selection (GS) on stripe rust, leaf rust, Fusarium head blight (FHB), and leaf spot in wheat, but none of them have assessed the effect of the reaction norm model that incorporated GE interactions. In addition, the prediction accuracy on common bunt has not previously been studied. Here, we investigated within-population prediction accuracies using the baseline M1 model and two reaction norm models (M2 and M3) with three random cross-validation (CV1, CV2, and CV0) schemes. Three Canadian spring wheat populations were evaluated in up to eight field environments and genotyped with 3158, 5732, and 23,795 polymorphic markers. The M3 model that incorporated GE interactions reduced residual variance by an average of 10.2% as compared with the main effect M2 model and increased prediction accuracies on average by 2–6%. In some traits, the M3 model increased prediction accuracies up to 54% as compared with the M2 model. The average prediction accuracies of the M3 model with CV1, CV2, and CV0 schemes varied from 0.02 to 0.48, from 0.25 to 0.84, and from 0.14 to 0.87, respectively. In both CV2 and CV0 schemes, stripe rust in all three populations, common bunt and leaf rust in two populations, as well as FHB severity, FHB index, and leaf spot in one population had high to very high (0.54–0.87) prediction accuracies. This is the first comprehensive genomic selection study on five major diseases in spring wheat.

## 1. Introduction

Leaf rust, stripe (yellow) rust, stem rust, common bunt, Fusarium head blight (FHB), and leaf spot complex (the blotch diseases) are the most common and economically important wheat diseases in Canada and across the rest of the world [1,2,3,4]. Leaf rust caused by *Puccinia triticina* f. sp. *tritici* is common in mild temperature and moist conditions [4], with varying levels of severity from year to year [5]. Stripe rust (*P. striiformis* f. sp. *tritici*) has been detected in western Canada every year since 2000, with serious epidemics reported in some parts in 2005, 2006, and 2011 [6]. Multiple stem rust (*Puccinia graminis* f. sp. *tritici*) epidemics were reported in the country in the early 1900s and from 1953 to 1955, which caused losses worth hundreds of millions of dollars [7]. Although the severity of the three rusts can be reduced through agronomic management practices and the application of foliar fungicides, the development and deployment of resistant cultivars is environmentally friendly and safer [8]. Common bunt (also known as stinking smut and covered smut) is caused by both *Tilletia tritici* (syn. *Tilletia caries*) and *T. laevis* (syn. *T. foetida*) [9], which can be seed-borne (smut spores on the seed) or soilborne. Infected plants are stunted, producing fewer and smaller spikes than normal plants, which ultimately reduces both grain yield and quality through the formation of black masses of spores (bunt balls) [10]. The replacement of grains with bunt balls not only reduces grain yield, but also affects quality due to undesirable odors in the flour that are not acceptable by wheat millers. The three strategies used to manage common bunt include seed treatments with appropriate fungicides, crop rotation to minimize the buildup of the pathogen, and planting resistant cultivars.

FHB, or scab, can be caused by multiple fungal species, with *Fusarium graminearum* (Schwabe) being the most destructive disease in parts of western Canada [11,12]. FHB affects grain yield, grain quality, and marketability in three ways. First, it causes premature bleaching of florets (spikes), which results in sterility and small white-to-pink shriveled seeds, which substantially reduce grain yield [13]. Second, FHB-infected kernels are of poor quality and are either rejected or downgraded due to the presence of discolored and shriveled seeds that substantially reduce grain price. Third, infected kernels may be contaminated with mycotoxins, such as deoxynivalenol (DON), nivalenol, and zearalenone, which are toxic to animals and humans [14]. FHB management methods include the use of appropriate fungicides, crop rotation, seed treatment, cultural practices (e.g., staggering planting time, increasing seeding rate, irrigation management), and planting resistant cultivars [15,16,17]. None of the management methods are effective on their own, and multiple methods must be used to reduce losses to FHB. Fungicide application to control FHB is suppressive rather than preventative or curative, and is not as effective in controlling FHB as compared with rusts and leaf spots, because timing the application of fungicide for FHB control is challenging due to the narrow application window [4]. Furthermore, most modern wheat cultivars have mutant reduced height alleles (*Rht-D1b* and/or *Rht-B1b*), which increase susceptibility to FHB by reducing anther extrusion [18,19,20]. Leaf spotting is one of the most prevalent diseases in wheat-growing areas in Canada and several other countries globally. In Canada, it is the second priority disease (Priority-2) caused by multiple pathogens: tan spot (*Pyrenophora tritici-repentis*), septoria leaf blotch complex (caused by *Phaeosphaeria nodorum*, *Mycosphaerella graminicola* and *Phaeosphaeria avenaria*), and spot blotch (*Cochliobolus sativus*) [21]. These pathogens commonly occur as complexes and can reduce test weight and grain yield by about 50%. High levels of leaf spot resistance require resistance to each of the causal species.

In Canada, new varieties (cultivars) should possess a combination of 30–40 target traits depending on the market class (https://grainscanada.gc.ca/en/grain-quality/grain-grading/wheat-classes.html; accessed 20 March 2022), which must include at least intermediate levels of resistance to the three rusts, FHB, and common bunt. Genetically resistant cultivars can be developed using conventional breeding methods, marker-assisted selection (MAS), and genomic selection (GS). MAS involves introgressing a few genes or major effect quantitative trait loci (QTL) from trait donors into elite genetic backgrounds, which is often challenging due to (i) the need to pyramid different sources of resistance into the same genetic background, and (ii) concerns associated with the durability of introgressed genes that regulate qualitative or vertical resistance against multiple races. Vertical resistance tends to be expressed from seedling to adult plant stages, but they lose their effectiveness over time due to changes in pathogen populations. On the other hand, quantitative resistance is a partial level of resistance controlled by multiple minor to moderate effect genes and/or QTL, which are more durable but require the pyramiding of multiple favorable alleles, which makes the suitability of MAS more challenging. GS is a promising alternative to MAS for predicting the most likely performance of lines by incorporating all available molecular marker information into a model to compute genomic estimated breeding values for selection [22,23,24,25].

Although numerous proofs of concept and pilot GS studies have been conducted in wheat to investigate its potential for improving multiple agronomic traits, yield components, and end-use quality traits in wheat, only a few studies have been conducted on diseases. The latter include some studies conducted to evaluate the predictive ability of FHB [26], stripe rust [27,28], rusts, and leaf spots [29,30,31] in spring wheat, FHB in winter wheat [32,33], rusts in durum wheat [34] and winter wheat [35,36,37,38], *Septoria tritici blotch* (STB) and tan spot in winter wheat [39,40,41], as well as FHB and STB in winter wheat [42,43,44]. Each of these studies reported highly variable prediction accuracies, but they neither evaluated all diseases on the same population nor compared the predictive ability of the different reaction norm models [45], which forms one of the bases in the present study. In addition, we are not aware of any study that investigated the predictive ability of GS in common bunt in wheat, which forms another basis in the present study.

The multiplicative reaction norm model [45] is one of the methods proposed to account for genotype × environment (GE) interactions that could improve prediction accuracies [46,47,48,49,50]. It partitions the phenotypic variance into genotypes (lines), molecular markers (genomics), environments (E), and GE interactions, and compares the predictive ability of the baseline model with the main effect and interaction models. In our previous study [51], we used the reaction norm models with three random cross-validation (CV) schemes in three Canadian spring wheat populations. Each population was evaluated for seven agronomic and grain characteristics in three to nine conventional and three to six organically managed field environments which were genotyped either with the wheat 90K SNP array or DArTseq technology. Our results from that study revealed highly variable prediction accuracies depending on the models, CV schemes, trait complexity, and genetic backgrounds. The objectives of this study were to (1) compare the variance components and heritability of FHB, common bunt, leaf rust, stripe rust, and leaf spot across three spring wheat populations, and (2) examine the predictive ability of three models and random cross-validation (CV) schemes across different genetic backgrounds and traits.

## 2. Materials and Methods

### 2.1. Germplasm and Phenotyping

The present study was conducted on 578 lines and cultivars (Table S1) that represented an association mapping panel of 203 lines and cultivars plus 2 recombinant inbred lines (RIL) populations derived from Attila × CDC Go (167 lines), and Peace × Carberry (208 lines). These three Canadian spring wheat populations were used in our previous study to compare the prediction accuracies of different models, and random CV schemes across seven agronomic and end-use quality traits [51]. The following cultivars were used as resistant and susceptible checks: (i) AC Barrie and AC Crystal as susceptible, and Lillian and Carberry as resistant checks in stripe rust nurseries; (ii) AC Barrie and Park as moderately susceptible to susceptible checks, and Peace and Carberry as moderately resistant to resistant checks in leaf rust nurseries; (iii) AC Barrie, Unity, and Glenlea as moderately susceptible checks, and Neepawa as a moderately resistant checks in the leaf spot nurseries; (iv) Laura as susceptible and McKenzie as a resistant checks in common bunt nurseries; and (v) CDC Teal as a susceptible check and 5602HR as moderately resistant checks in FHB nurseries.

The association mapping panel (hereafter referred to as BVC) consisted of 183 Canadian spring wheat cultivars and 20 unregistered spring wheat lines [52]. It was evaluated at eight environments (site × year combinations) for reaction to stripe rust near Creston, British Columbia (49.06° N, 116.31° W) in 2018 and 2020, at the Lethbridge Research and Development Centre (Lethbridge RDC), Alberta (49.7° N, 112.83° W) between 2016 and 2019, and at the University of Alberta South Campus, Edmonton, Alberta (53°19′ N, 113°35′ W) in 2016 and 2018. The BVC panel was evaluated for reactions to leaf rust at four environments at the University of Alberta South Campus in 2017 and 2020, and the Morden Research and Development Centre (Morden RDC), Manitoba, in 2019 and 2020. For both stripe and leaf rusts, seeds of each cultivar and check were planted either in hills or 1 m long rows with a spacing of 25 cm between hills or rows in disease screening field nurseries using a randomized incomplete block design with two replications. The susceptible checks were used as spreader rows, and were planted every five rows. Inoculum preparation and application were carried out as described in a previous study [53]. At each site (location), except Creston for stripe rust that was based on natural infection, we collected urediniospores of the prevalent multi-race mixture from infected plants in the previous year and froze them in −80 °C in vials until needed in June of the following year. Urediniospores were removed from the freezer, allowed to rehydrate at room temperature, suspended in light mineral oil (Soltrol 170, Chevron Phillips Chemical Co., Woodlands, TX, USA) at a concentration of 1 mL urediniospores and 2 L of Soltrol 170, and sprayed on the leaves of the spreader rows at an early tillering stage in the early evenings using a low-volume hand sprayer. The spray application was repeated three times at an interval of three days. Subsequently, urediniospores that developed on the spreader rows were windblown to the test cultivars and lines to provide infection. Plants were irrigated three times a week using either overhead sprinklers or Cadman Irrigations travellers with Briggs booms.

The BVC panel was evaluated for its reaction to leaf spot for three years at the University of Alberta South Campus in 2016, 2017, and 2018. *P. tritici-repentis*, which causes tan spot, is the most common leaf spotting disease in all wheat classes. Disease epidemics was initiated by spraying spreader rows of susceptible checks (AC Barrie, Unity, and Glenlea) with a spore suspension that consisted of an equal mixture of two isolates (AB7-2 and AB50-2) that belong to race 1 of *P. tritici-repentis* (Ptr). These two isolates contain the *ToxA* gene [54] and belong to Race 1, which is the most common race in Alberta [55]. Although both AB7-2 and AB50-2 isolates cause tan spot disease, we scored disease severity as leaf spot because tan spot infection in wheat fields occurs in association with septoria blotch and spot blotch, which all produce similar leaf lesions that are difficult to visually distinguish without laboratory analysis [56]. For both rusts and leaf spot, disease severity was recorded using a modified Cobb scale [57] on a scale of 1 (no visible sign or symptom = resistant) to 9 (leaf area covered with spores = highly susceptible). Such ratings were performed when the susceptible and resistant checks showed contrasting reactions (susceptible check had many pustules/lesions, the moderate checks had fewer pustules/lesions, and the resistant check had few or no pustules/lesions).

The BVC panel was evaluated for its reaction to common bunt at the University of Alberta South Campus in 2016 and 2017, as described in a previous study [58]. The screening involved treating seeds of each cultivar with an equal mixture of common bunt race L-16 of *T. laevis* and race T-19 of *T. tritici* spores. We then removed the excess inoculum by shaking the seed over a fine-mesh sieve and planted them in a randomized incomplete block design with two replications. At the dough stage, all spikes of each cultivar were examined for common bunt infection and recorded on a scale of 1 to 9, in the same manner as rusts and leaf spots. The reaction to FHB was evaluated at six environments at the Elora Research Station, Pilkington, Ontario in 2017, at the Morden RDC from 2017 to 2019 and 2021, and the Ian N. Morrison Research Farm, University of Manitoba, Carman, Manitoba in 2020. The inoculum suspension consisted of four *F. graminearum* isolates that belong to the 3-ADON chemotype (HSW-15-39 and HSW-15-87) and the 15-ADON chemotype (HSW-15-27 and HSW-15-57), as described in a previous study [12]. Briefly, the suspension was prepared by mixing an equal amount of each isolate with sterile water and Tween 20 and applied directly to wheat spikes using a backpack sprayer. The sprays were performed twice at Morden and Carman, and three times at the Elora Research Station, starting when the earliest lines reached a 4–5 leaf stage. The inoculated plants were irrigated three times a week using either an overhead mist irrigation system (Elora and Carman) or Cadman Irrigations travellers with Briggs booms (Morden). Visual FHB rating was performed 18–21 days after the first spray inoculation by examining infected spikes (disease incidence) and infected spikelets (disease severity) on a scale of 1 to 9 in some environments and 0–100% at other environments, as described above. To get the same FHB rating across all environments, however, we converted the percentages scores into the 1 to 9 scale and then calculated visual rating index (VRI) in percentages by multiplying FHB incidence and FHB severity.

The ACG population was evaluated for reactions to leaf rust, leaf spot, and common bunt at three environments at the University of Alberta South Campus Research Station from 2012 to 2014, as described in our previous study [59]. The ACG population was also evaluated for reactions to stripe rust at three environments at the Lethbridge RDC in 2013, at Creston in 2014, and at the Ellerslie research station, Edmonton, Alberta, in 2015. In both Creston and Lethbridge, plots consisted of a single 1 m long row per line spaced 25 cm between plants in a randomized complete block design with two to three replicates depending on seed availability. At Ellerslie, we grew hill-plots of ten seeds per line spaced 25 cm apart, with a similar experimental design. The PAC population was evaluated for stripe rust at eight environments at Creston in 2016, at the University of Alberta South Campus Research Station in 2016, 2018, and 2019, and at Lethbridge RDC from 2016 to 2019. Leaf rust was evaluated at three environments at the University of Alberta South Campus Research Station in 2017 (nursery and field) and 2020 (nursery), whereas common bunt was evaluated at the same location three times in 2017 (nursery and field) and 2018 (nursery). Leaf spot was evaluated at four environments at the University of Alberta South Campus Research Station in 2016 (nursery), 2017 (field and nursery), and 2018 (nursery). The experimental design, isolates, inoculum preparation, inoculum application, and disease ratings were the same as the BVC population described above.

### 2.2. DNA Extraction and Genotyping

Genomic DNA was extracted following a modified cetyl trimethyl ammonium bromide (CTAB) method [60], and its quality was checked by running an aliquot onto 0.8% agarose gel with SYBR Safe DNA Gel Stain. The DNA concentration was assessed with a NanoDrop ND-1000 Spectrophotometer (Thermo Scientific, Waltham, MA, USA), normalized to approximately 100 ng μL^−1^, and shipped to service laboratories for genotyping. DNA samples from both the BVC and ACG populations were genotyped with the wheat 90K iSelect array [61] at the University of Saskatchewan Wheat Genomics lab, Saskatoon, Canada, whereas the PAC population was genotyped with DArTseq technology (https://www.diversityarrays.com/; accessed 20 March 2022). The DArTseq technology generated a total of 36,626 markers, of which 22,741 were SilicoDArT markers (present vs. absent variation) and the remaining 13,885 were SNPs. We also genotyped the ACG and PAC RIL populations with a few functional markers linked to the photoperiod response (*Ppd-B1* and *Ppd-D1*) [62], vernalization response (*Vrn-A1* and *Vrn-B1*) [63], and/or *Rht-B1* [64] genes at the Agricultural Genomics and Proteomics Lab, University of Alberta, Edmonton, Canada, as described in our previous studies [63,65,66]. In the BVC population, we excluded markers that had >20% missing data points, and those with minor allele frequencies below 5%. In the RIL populations, we excluded all markers that had >20% missing data, were monomorphic between parents, and had high segregation distortion at *p* < 0.01. We finally retained a total of 23,795 SNPs in the BVC, 5732 markers (3840 SilicoDArT and 1892 SNPs) in the PAC, and 3158 SNPs in the ACG population for subsequent analyses (Table S2).

### 2.3. Data Analyses

All data analyses were performed as described in our previous study [51]. Briefly, we computed best linear unbiased estimators (BLUEs) as adjusted means [34,39] using Multi Environment Trial Analysis in R (META-R) v6.04 (https://hdl.handle.net/11529/10201; accessed 20 March 2022). Broad-sense heritability (H^2^) across all environments was computed as follows:

$$H^{2}=\frac{\sigma^{2}g}{\sigma^{2}g+\frac{\sigma^{2}e+{\;\sigma }^{2}\mathrm{ge}}{\mathrm{Env}}+\frac{\sigma^{2}\varepsilon }{\mathrm{Env}\;x\;\mathrm{Rep}}}$$

where σ^2^_g_, σ^2^_e_, σ^2^_ge_, and σ^2^_ε_ are the genotypic, environmental, genotype-by-environment interaction, and residual (error) variance components, respectively, whereas *Env* and *Rep* are the number of environments and the average number of replicates within each environment, respectively. The disease ratings were analyzed separately for each environment and combined across all environments by considering genotypes (L) as a fixed effect and environment (E), replication, and GE interaction as random effects. Pearson correlations and coefficient of determination (*R^2^*) and different types of graphs were generated using both Minitab v.14 (https://www.minitab.com; accessed on 20 March 2022) and JMP v16 (www.jmp.com; accessed on 20 March 2022) statistical software’s. We computed the first three principal components from the genotype data of each population in TASSEL v5.2.72 [67], used them as covariates in the GS prediction models, and plotted them for visual examination of within-population structure in CurlyWhirly v1.21.08.16 (https://ics.hutton.ac.uk/curlywhirly/; accessed 20 March 2022).

We evaluated the predictive ability of pairwise combinations of the baseline model and two reaction norm models and three CV schemes [45,68], as described in our previous study [51]. Briefly, the baseline M1 model considers the response of the *j*th wheat line in the *i*th environment ($y_{ij})\;$as a function of a random effect model that accounts for the effects of the environment ($E_{i}$), the line ($L_{j})$ plus a residual variance ($\varepsilon_{ij}$) as follows:

(1) $$y_{ij}=\mu +E_{i}+L_{j}+\varepsilon_{ij}$$

where μ is an intercept, $E_{i}\overset{iid}{\sim }N\left(0,\;\sigma_{E}^{2}\right)$ is the random effect of the *i*th environment, $L_{j}\overset{iid}{\sim }N\left(0,\;\sigma_{L}^{2}\right)$ is the random effect of the *j*th line, $\varepsilon_{ij}\overset{iid}{\sim }N\left(0,\sigma_{\varepsilon }^{2}\right)$ is a residual, and N(·, ·) stands for a normally distributed random variable that is independent and identically distributed (*iid*). The M1 model assumes the effects of the lines as independent with no borrowing of information among lines. The main effect M2 model is an extension of the M1 model, which adds the random effect of molecular markers or genomic ($g_{j})$ as follows:

(2) $$y_{ij}=\mu +E_{i}+L_{j}+g_{j}+e_{ij}$$

The M2 model allows the borrowing of information among lines that enable the prediction of untested genotypes. The third model (M3) extends the M2 model (Equation (2) above) by including genomic and environment interaction effects ($Eg_{ij}$) as follows:

(3) $$y_{ij}=\mu +E_{i}+L_{j}+g_{j}+Eg_{ij}+e_{ij}$$

Notably, in Equation (3), the interaction term $Eg_{ij\;}$ approximates the true interaction of the *i*th line with the *i*th environment and conceptually includes all the first pairwise interactions between each genotype at each environment. The wheat lines are related; therefore, the M3 model allows the borrowing of information between the lines to predict line performance in environments where the lines are not observed. Each of these three models was assessed using three prediction scenarios that mimicked (i) predicting the performance of 20% of wheat lines that have not been evaluated in any of the environments (CV1), (ii) predicting the performance of 20% of lines that were evaluated in some environments (CV2) but not in others, which used phenotypes of the same line at different environments as part of the training set, and (iii) predicting the performance of all lines in an unobserved environment using the remaining environments as a training set (CV0), which was conducted in a leave-one-out fashion. The CV0 scheme computed the correlation between predicted and observed values within each environment only once with no random process involved to assign lines into folds. All genome-wide prediction analyses were performed using imputed marker data in R and the Bayesian Generalized Linear Regression (BGLR) package, as described elsewhere [69].

## 3. Results

### 3.1. Reaction to Diseases

**Figure 1:**
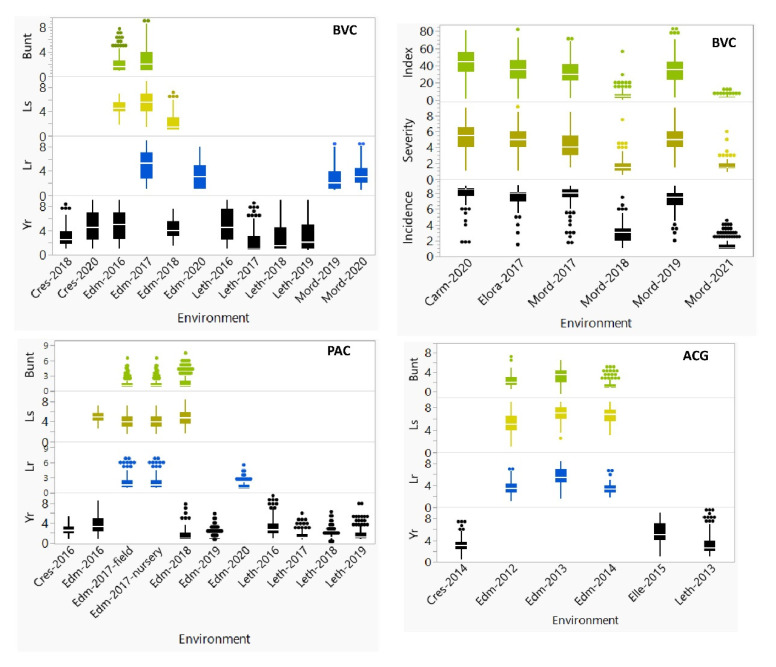
Box plots of the best linear unbiased estimators (BLUEs) of Fusarium head blight (FHB), stripe rust (Yr), leaf rust (Lr), leaf spot (Ls), and common bunt (bunt) in three spring wheat populations evaluated in field conditions. All diseases were rated on a 1 to 9 scale, except the FHB index in percentage. Both ACG and PAC are recombinant inbred lines derived from Attila × CDC Go and Peace × Carberry, respectively, whereas BVC is an association mapping panel of diverse spring wheat cultivars and lines. The environments on the *x*-axis start with a prefix for each site (Cres: Creston station in British Columbia; Edm: the University of Alberta South Campus in Edmonton; Elle: the Ellerslie research station in Edmonton; Leth: Lethbridge Research and Development Centre; Carm: the University of Manitoba Research Farm in Carman; Mord: Morden Research and Development Centre), followed by the year of the experiment.

**Figure 2:**
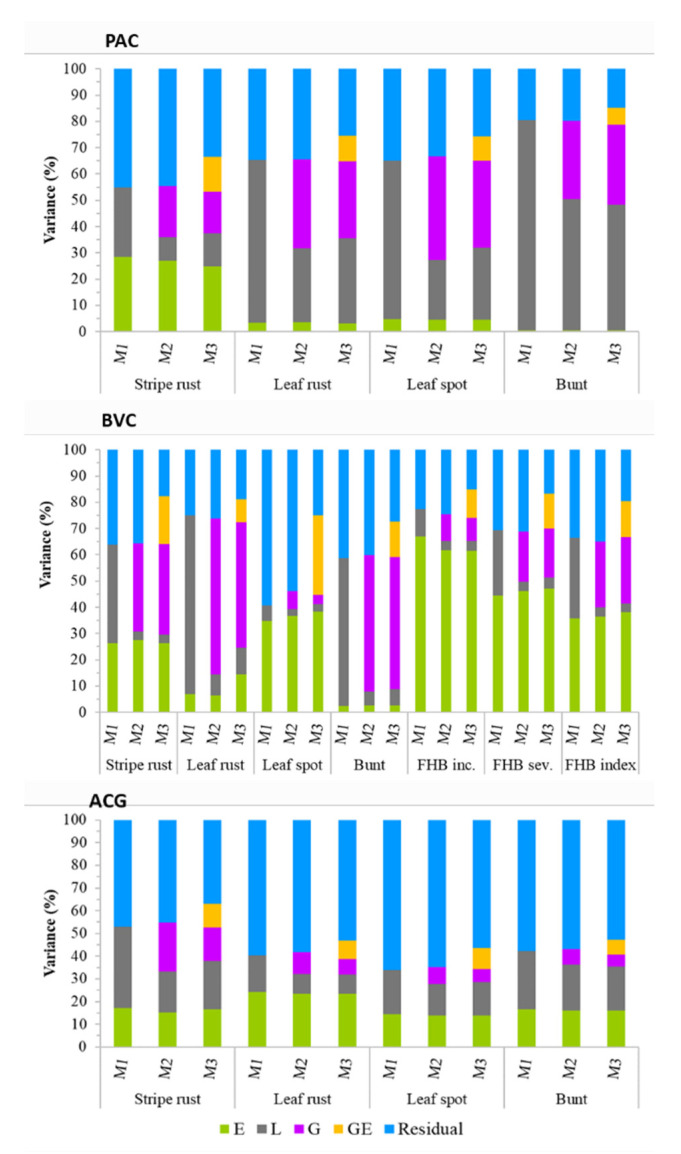
Partitioning of total variance into environments (E), genotypes (L), molecular markers (G), interactions between genotypes and environments (GE), and residual components using the baseline M1 model, the main effect M2 model, and the M3 model that incorporated GE interactions. See Table S4 for details.

Figure 1 summarizes disease scores based on individual environments, which revealed highly variable reactions depending on the genetic background, the environment, and the type of disease. Genotypes (lines) within each of the three populations showed significant (*p* < 0.05) differences for all diseases (Table S3). Table 1 summarizes the disease scores, correlations among environments, and broad-sense heritability computed across all environments. Stripe rust, leaf rust, leaf spot, common bunt, FHB incidence, and severity scores in the BVC panel evaluated between two and eight environments varied from 1.0 to 9.0 in the individual environments and from 1.0 to 8.1 combined across all environments. The FHB index across six field environments in Ontario and Manitoba varied from 0.5% to 82.1% on individual environments (Table 1), and from 3.7% to 49.6% averaged across all six environments. In the PAC population, stripe rust, leaf rust, leaf spot, and common bunt scores in 3–8 individual environments varied from 1.0 to 9.0, from 1.0 to 6.9, from 1.4 to 8.3, and from 1.0 to 7.5, respectively. Averaged across all environments, the RILs in the PAC population had a disease score of 1.1–5.7 for stripe rust, 1.0–5.8 for leaf rust, 2.3–7.1 for leaf spot, and 1.0–6.0 for common bunt. Disease scores in the ACG RIL population evaluated across three environments varied from 1.5 to 7.9 for stripe rust, from 1.9 to 6.1 for leaf rust, from 3.0 to 8.7 for leaf spot, and from 1.0 to 4.9 for common bunt, respectively. Broad-sense heritability in each population varied from 0.44 to 0.62 in the ACG, from 0.22 to 0.89 in the BVC, and from 0.72 to 0.90 in the PAC populations. Leaf spot in the BVC panel showed the lowest broad-sense heritability, which was due to the high environmental variance observed in this population (34.8–38.3%) as compared with the PAC (4.5–4.7%) and ACG (13.8–14.3%) populations (Figure 2). The second lowest heritability was observed for FHB incidence in the BVC population, which was also due to high environmental variance (61.5–67.1%) as compared with FHB severity (44.4–47.0%) and index (35.9–38.2%).

**Figure 3:**
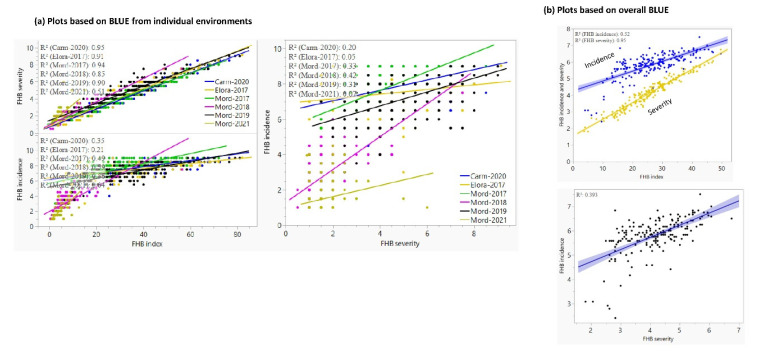
Regression plots of Fusarium head blight (FHB) incidence, severity, and index based on (**a**) individual environments, and (**b**) all six environments. The plots showed low *R^2^* between incidence and severity (0.05–0.42), low to moderate R^2^ between incidence and index (0.21–0.64), and high to very high R^2^ between severity and index (0.51–0.95). The environments are named using the site (Elora, Mord for Morden, and Carm for Carman) and the year of the experiment. Note that FHB incidence showed a lower *R*^2^ value with FHB severity and index due to its greater environmental variances summarized in Table S3 and Figure 2.

The correlation between pairs of environments within the same disease and the same population varied from 0.07 to 1.00 (Table 1). Low to moderate correlations were observed among the six environments used for evaluating FHB incidence (0.12–0.56), severity (0.11–0.67), and index (0.10–0.68) (Figure S1). The correlation between pairs of traits recorded in each population varied from −0.01 to 0.38 in the ACG, from −0.24 to 0.30 in the PAC, and from −0.11 to 0.97 in the BVC population. In the overall mean disease scores across all environments, the three highest correlations were observed between FHB severity and FHB index (r = 0.97, *p* < 0.01), FHB incidence and FHB index (r = 0.72, *p* < 0.01), and FHB incidence and FHB severity (r = 0.63, *p* < 0.01) in the BVC population (Figure 3 and Figure S2). The high correlation between FHB severity and FHB index was also evident between pairs of the six environments with correlations and coefficients of determination varying from 0.71 to 0.97 (Figure S2) and 0.51 to 0.96 (Figure 3), respectively. The correlation between FHB incidence and severity and between incidence and index across the six environments varied from 0.22 to 0.65 and from 0.45 to 0.80, respectively. All disease scores averaged across all environments showed a continuous distribution in all three populations (Figure S3). A plot of the first two PCs from PCA analyses revealed some level of population structure within both the BVC and the ACG RIL populations, but not within the PAC population (Figure S4).

### 3.2. Partitioning of Variance Components

The proportion of variances due to environments, genotypes (lines), molecular markers (genomics), GE interactions, and residual (error or unexplained) components differing depending on the prediction models, traits, and genetic backgrounds (Figure 2, Table S4). Environmental variances across all populations and diseases varied from 0.5% for common bunt in the PAC population to 78.6% for FHB incidence in the BVC panel, with an overall average of 24.1%. Genotypic variance varied from 2.0% for FHB incidence in the BVC panel to 80.0% for common bunt in the PAC population, and the overall average was 21.2%. The effects of genomics on variance components were estimated in both M2 and M3 models, which varied from 3.4% for leaf spot to 59.4% for leaf rust in the BVC population, with an overall average of 22.2%. The variances due to GE interactions computed in the M3 model varied from 5.9% for FHB incidence to 30.2% for leaf spot in the BVC panel, with an overall average of 11.6%. Residual variance varied from 9.6% to 66.2% with an overall average of 35.9%. Residual variance was much greater in the ACG population (range: 36.9–66.2%, average = 54.6%) than the BVC (range: 9.6–59.3%, average = 28.4%) and PAC (range: 14.9–45.0%, average = 30.5%) populations. Averages across all diseases and models, environment, genotype, genomics, GE, and residual variances within each population varied from 8.8% to 36.5%, from 13.0% to 38.3%, from 9.7% to 28.9%, from 8.6% to 14.5%, and from 28.4% to 54.6%, respectively. The highest environmental variance was observed for FHB incidence, severity, and index in the BVC panel, whereas the highest residual variances were observed in the ACG population (Figure 2), which generally agreed with the broad-sense heritability (Table 1). In contrast to the positive correlation between heritability and genotypic variance (r = 0.57, *p* < 0.01), both residual and environmental variances showed a significant negative correlation (−0.70 ≤ r ≤ −0.30, *p* < 0.01) with heritability. As compared with the main effect M2 model, the inclusion of GE interactions in the M3 model reduced the residual variances on average by 10.4% (range 3.9–28.8%). However, the residual variance per trait still accounted for an overall average of 28.7% (range 9.6–56.3%) in the M3 model, with the ACG population showing the highest average residual variance at 49.8% (range 36.9–56.3%).

### 3.3. Comparison of Prediction Models

**Figure 4:**
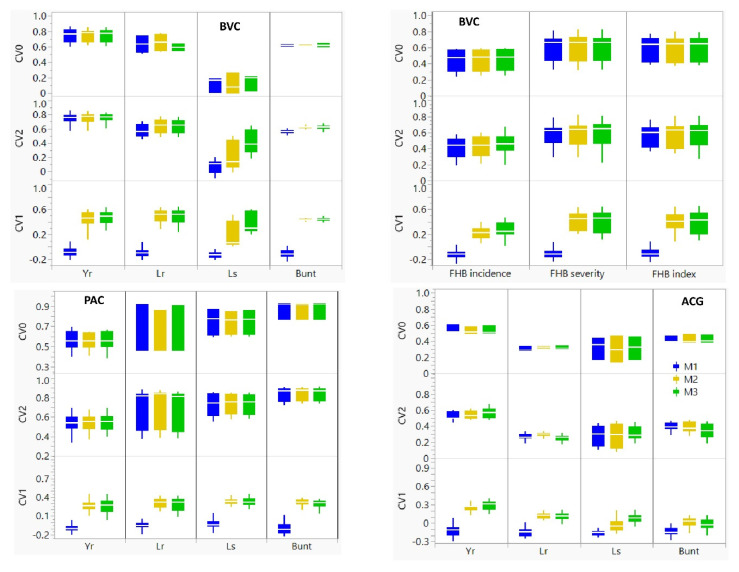
Comparisons of prediction accuracies of three random cross–validation schemes (CV0, CV1, and CV2) and three models (M1, M2, and M3). CV0, CV1, and CV2 represent predicting the entire environment, the performance of newly developed lines, and sparse testing, respectively. M1, M3, and M3 represent the baseline model, the main effect reaction norm model, and the model that incorporated GE interactions, respectively. Trait initialisms are as follows: stripe rust (Yr), leaf rust (Lr), leaf spot complex (Ls), common bunt (bunt), and Fusarium head blight (FHB). See Table S5 for details.

We compared the predictive ability of the baseline M1 model that utilizes only phenotype data with the main effect M2 model that adds molecular markers to the M1 model, and the M3 model that incorporated GE interactions to the M2 model (Figure 4, Table 2 and Table S5). In the absence of molecular markers, the M1 model produced negative or close to zero prediction accuracies for all diseases and populations when it was used with the CV1 scheme (range −0.16 to −0.03, average −0.11), which suggested the failure of the model to predictive disease reaction of presumably ‘newly developed lines’. The M1 model showed highly variable accuracies when it was used with the CV2 (range: 0.07 to 0.84, average 0.51) and CV0 (range: 0.11 to 0.87, average 0.55) schemes, which was due to the inclusion of some phenotype records of the same lines at some environments. When molecular markers were incorporated in the M2 model, the average prediction accuracies per trait with the CV1, CV2, and CV0 schemes varied from 0.02 to 0.49, from 0.21 to 0.84, and from 0.11 to 0.87, respectively. The overall average prediction accuracies of all diseases and populations obtained when the M2 model was used with the CV1, CV2, and CV0 schemes were 0.28, 0.54, and 0.55, respectively. The prediction accuracies of the M3 model with the CV1, CV2, and CV0 schemes varied from 0.02 to 0.48, from 0.25 to 0.84, and from 0.14 to 0.87, respectively. The average prediction accuracies with the M3 model were 0.30 for CV1 and 0.55 for both CV2 and CV0.

Overall, prediction accuracies obtained with the M1 model with the CV1 and CV2 schemes were significantly smaller (*p* < 0.05) than both the M2 and M3 models (Table S5D). The most noticeable change in prediction accuracies in all diseases and populations was observed when the CV1 scheme was used with the M2 and M3 models that increased accuracies by averages of 208% and 199% over the baseline M1 model, respectively. We found statistically significant differences in the prediction accuracies between the M2 and M3 models in the BVC, but not in both the PAC and ACG populations, regardless of the CV schemes. However, the M3 model increased prediction accuracies over the M2 model by an average of 6% at CV1 and by 2% both at CV2 and CV0 schemes. In some traits, the M3 model increased prediction accuracies by 4–54% as compared with the M2 model, which included leaf spot in both the BVC and ACG populations at both the CV1 and CV2 schemes and FHB incidence and index in the BVC population (Table 2). The similarities in the prediction accuracies of the M2 and M3 are evident from the high coefficients of determination in most traits (Figure S5a). In cases when one model performed better than the other, however, the coefficient of determination between the M2 and M3 models showed an erratic pattern, which included FHB incidence (0.82–1.00), FHB index (0.90–0.99), and leaf spot (0.59–0.95) in the BVC population, leaf spot in ACG (0.36–0.99) and PAC (0.83–1.00), and leaf rust in the ACG (0.44–0.82) and PAC (0.90–1.00) population (Figure S5b).

**Figure 5:**
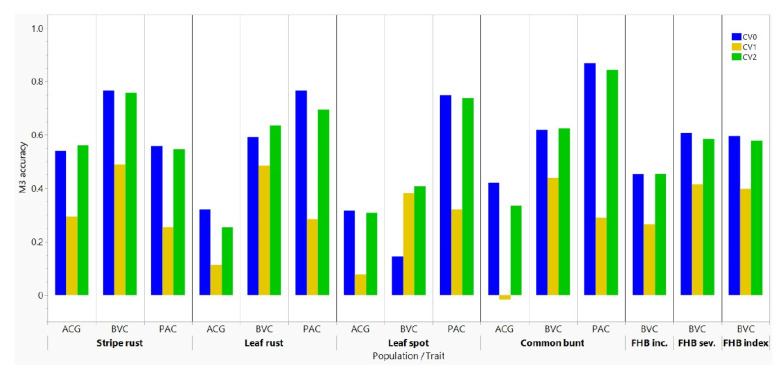
Comparisons of average prediction accuracies using the M3 model with the CV0, CV1, and CV2 random cross-validation schemes that represent predicting the entire environment, the performance of newly developed lines, and sparse testing, respectively. See Table S5 for details.

We compared the average prediction accuracies of the five diseases to determine if some environments provided better predictions than others (Figure S6). Although most environments showed consistent prediction accuracies regardless of the genetic background and the trait, some environments displayed lower accuracies. For example, Creston for stripe rust resistance in both BVC and PAC populations, and Morden in 2017 and 2021 for FHB resistance in the BVC population gave lower accuracies as compared with other environments. Using the results of the M3 model, we also compared the prediction accuracies of all four to seven traits per population regardless of the environments (Figure 5, Table 2). The predictive ability of the M3 model with both the CV0 and CV2 schemes for common bunt and leaf rust was very high in the PAC (0.70–0.87) population, high in the BVC (0.59–0.64), and low to moderate (0.25–0.42) in the ACG population. For leaf spot, the M3 model with the CV2 and CV0 schemes provided high accuracies (0.74–0.75) in the PAC population and low to moderate (0.14–0.42) in the BVC and ACG populations. The prediction accuracies of FHB severity and index in the BVC populations were similar, which varied from 0.40 to 0.41 at CV1 and from 0.60 to 0.63 at CV2 and CV0. The prediction accuracies for FHB incidence were 0.27 at CV1 and 0.45 both at CV2 and CV0, which were 24–36% smaller than that of FHB severity and index. Overall, the ACG population had low prediction accuracies for common bunt, leaf rust, and leaf spot, regardless of the CV schemes (Figure 5).

## 4. Discussion

### 4.1. Comparisons of Prediction Models

At least 83 stripe rust, 80 leaf rust, and 15 common bunt resistance genes have been reported in wheat and its relatives [70,71,72], which regulate the qualitative inheritance of these diseases. However, quantitative resistance has frequently been reported as the predominant form of resistance in crops [73,74], which is regulated by multiple QTLs of minor effects, highly polygenic, and characterized by a continuous phenotypic distribution. The continuous distribution of all five diseases evaluated in the present study was evident in all three populations (Figure S3) regardless of the genetic backgrounds (biparental RILs vs. diverse panel of cultivars from multiple wheat classes), which suggest the lack of a major gene and major effect QTLs. Previous genome-wide association analysis and standard QTL mapping studies conducted in the BVC, PAC, and ACG populations detected only a few minor to moderate effect QTLs that individually accounted for <20% of the phenotypic variance [59,75,76]. In such cases, therefore, genome-wide prediction outperformed MAS, which produced intermediate to high prediction accuracies (0.4–0.9), as compared with the lower values (<0.30) observed for MAS-based models [77]. Although prediction accuracies obtained in the present study differed depending on the trait, genetic background, model, and CV schemes (Table 2, Figure 4), most results are highly encouraging for implementing large-scale GS in Canadian spring wheat.

The incorporation of GE interactions in the M3 model has shown inconsistent results for agronomic and end-use quality traits in the literature, with some studies reporting higher prediction accuracies over the main effect M2 model [45,46,49,78,79,80], whereas others found no advantage at all [81]. Our results for the five diseases recorded in the BVC, PAC, and ACG populations revealed statistically significant differences between the M2 and M3 models in the BVC, but not within the ACG and PAC populations (Table S5c), which partly agrees with Juliana et al. [81]. The M3 model had three advantages: (a) it reduced residual variance per trait by 3.9–28.8% as compared with the M2 model; (b) it increased prediction accuracies by an average of 2–6%; and (c) we found a few cases where the M3 model increased prediction accuracies by up to 54% as compared with the M2 model.

GS predicts the most likely performance of lines using three scenarios that are widely used by plant breeders. In the CV1 scheme, we aimed to predict the performance of newly developed lines that have been genotyped with genome-wide markers but not phenotyped at any environment, which help breeder not only in facilitating the time of cultivar development, but also in reducing costs associated with seed multiplication, land preparation, and phenotyping. We used the CV1 scheme by hiding the phenotype data of 20% of randomly sampled lines as a testing set and the remaining 80% of them used as a training set, which was evaluated for up to 80 combinations of environments and iterations (Table S5A). The average prediction accuracies obtained using the CV1 scheme and the M3 model varied from 0.02 to 0.49, which is indicative of a lower probability in successfully implementing large-scale GS to predict the performance of newly developed lines without any field testing. On the other hand, the prediction accuracies of the M3 model with the CV2 scheme varied from 0.25 to 0.84, which suggests the high potential of implementing large-scale GS in developing disease resistance spring wheat cultivars using a sparse testing (incomplete field trials) design where some lines are evaluated in some environments, but not in others. Most of the prediction accuracies obtained when the CV2 scheme was used with the M3 model were ≥0.55, which included stripe rust (0.55–0.76) in all three populations, common bunt (0.62–0.84) and leaf rust (0.64–0.70) in the BVC and PAC populations, FHB severity (0.62) and FHB index (0.63) in the BVC population, and leaf spot (0.74) in the PAC population, which agree with other studies [78,82]. In the CV0 scheme, the main interest was to predict disease resistance for the future environment by leaving one environment out [47,78,83], which produced similar prediction accuracies to the CV2 scheme (Table 2).

### 4.2. Prediction Accuracies among Traits

Prediction accuracies obtained for common bunt when both CV0 and CV2 schemes were used along with the M3 model produced high accuracies in the BVC (0.62) and very high accuracies in the PAC (0.84–0.87) populations (Table 2). For the ACG population, prediction accuracies for common bunt were moderate (0.39 to 0.48), which agrees with the 0.40 accuracy reported for Karnal bunt (*Tilletia indica*) in two wheat RIL populations [84]. Differences in trait heritability, population size, and marker density may have contributed to the observed differences in prediction accuracies [85,86]. For example, broad-sense heritability was greater in both the BVC (0.69) and PAC (0.90) populations (Table 1) as compared with the ACG population (0.52). Population size in both BVC (203) and PAC (208) was greater than the ACG (167) population. Similarly, the marker density in the BVC population was 23793 SNPs, which was over four-fold and seven-fold greater than the 5731 markers in the PAC population and 3158 markers in the ACG population, respectively. The parents used in the ACG and PAC populations are included in the BVC populations. Our prediction accuracies for common bunt in the BVC and PAC population were 36% and 53% greater than the accuracies reported for Karnal bunt, respectively, which is very encouraging for wheat breeders to implement large-scale GS for developing common bunt resistant germplasm in western Canada. As far as we are aware, this is the first study to report the genome-wide predictive ability of different models and CV schemes for common bunt in wheat.

The prediction accuracies for both FHB severity and FHB index obtained when we used the M3 model with CV1 (0.40–0.41), CV2 (0.62–0.63), and CV0 (0.60–0.61) were 24–36% greater than those for FHB incidence (Table 2), which disagrees with accuracies reported in previous studies in spring wheat [26] and winter wheat [32]. Dong and colleagues [26] assessed the potential for genomic selection to improve FHB resistance using 170 spring wheat cultivars and elite lines adapted to the Pacific Northwest, evaluated in three field nurseries and one greenhouse, and genotyped with 10,101 SNPs selected out of the Wheat 90K SNP array. Using training and testing population sizes of 80% and 20%, respectively, the authors reported a higher average prediction accuracy for FHB incidence (0.63) than FHB severity (0.41). Using FHB phenotype data of 273 breeding lines from 18 winter wheat breeding programs in the United States, 3 prediction models, and 4500 SNPs, Arruda and colleagues [32] reported higher prediction accuracies for FHB incidence (0.60–0.63) than FHB severity (0.40–0.48). As shown in Figure 2, we observed much greater environmental variances for all three FHB-related traits than the four other diseases, with the highest being for FHB incidence. The percentage of phenotypic variance explained by the molecular markers was also much smaller for FHB incidence (8.9–10.2%) as compared with FHB severity (18.6–19.2%) and FHB index (25.0–25.3%), which may have reduced the predictive ability for incidence. In addition, broad-sense heritability for FHB incidence (0.29) was nearly half of that of severity (0.58) and index (0.50). A previous genomic selection study in winter wheat was conducted on 322 lines originated from 15 public and 3 private breeding programs across the eastern United States and Canada [87]. Using data from the U.S. cooperative FHB winter nurseries collected between 2008 and 2010, the authors reported highly variable average prediction accuracies for FHB incidence and severity, which ranged from 0.03 to 0.64 with the CV1 and from −0.12 to 0.61 with the CV2 schemes. Their two best models (Random Forest and Reproducing Kernel Hilbert Spaces) produced prediction accuracies for FHB incidence and severity that varied from 0.12 to 0.62 in the CV2 schemes. Our prediction accuracies for FHB severity and index in spring wheat agreed with the 0.58 average accuracy reported in European winter wheat [88], and were intermediate as compared with accuracies reported in other winter wheat populations evaluated at four environments in Germany (0.21 to 0.77) [43]. 

The average prediction accuracies for stripe rust using the M3 model with CV1, CV2, and CV0 schemes in our three spring wheat populations varied from 0.26 to 0.49, from 0.55 to 0.76, and from 0.54 to 0.77, respectively (Table 2). The predictive abilities for stripe rust with the CV2 and CV0 schemes were greater than the 0.26–0.33 accuracies reported in wheat landraces from Afghanistan [28], the 0.16 to 0.21 accuracies reported in hybrid winter wheat [36], and the 0.33–0.44 accuracies reported in another winter wheat landraces from the Australian Cereals Collection [37]. Muleta et al. [27] evaluated spring wheat accessions from the USDA-ARS National Small Grains and Potato Germplasm Research Unit and reported highly variable accuracies for stripe rust that ranged from 0.45 to 0.79. Using five wheat populations from the International Maize and Wheat Improvement Center (CIMMYT) evaluated in two environments, Ornella et al. [29] reported accuracies for stripe rust that varied from 0.12 to 0.63. Merrick et al. [38] reported accuracies that reached up to 0.72 for stripe rust severity in winter wheat.

For leaf rust, the prediction accuracies obtained when we used the M3 model with both CV2 and CV0 schemes were smaller (0.25–0.33) in the ACG population than the BVC (0.59–0.64) and PAC (0.70–0.77) populations (Table 2), which may be due to the differences in heritability, population size, and marker density discussed above. Broad-sense heritability for leaf rust was lower in the ACG (0.44) and higher in both the PAC (0.82) and BVC (0.78) populations (Table 1). Our results for both BVC and PAC populations were greater than the 0.43–0.50 accuracies reported in hybrid winter wheat [36], the 0.33–0.35 accuracies reported in winter wheat landraces from the Australian Cereals Collection [37], and the 0.10–0.38 accuracies reported for wheat landraces from Afghanistan [28]. The prediction accuracies for leaf spot obtained using the M3 model with the CV2 and CV0 schemes were inconsistent depending on the genetic background (0.14–0.41 in the BVC, 0.31–0.32 in the ACG, and 0.74–0.75 in the PAC populations). The prediction accuracies that we found for leaf spot in the PAC population were greater than the 0.45 to 0.66 reported in two populations from the International Bread Wheat Screening Nurseries [30] and the 0.40–0.42 mean accuracies reported in European winter wheat [41].

## 5. Conclusions

We compared prediction accuracies of the baseline model with two reaction norm models (M2 and M3) across five diseases recorded in three Canadian spring wheat populations. We found statistically significant differences in prediction accuracies among the three models in all three populations, but a significant difference between the M2 and M3 models was observed only in the association mapping panel. However, the M3 model that incorporated GE interactions showed some obvious advantages over the main effect M2 model, including increasing accuracies for traits up to 54% (2–6% on average per trait–population combination) as well as accounting for an overall average of 10.2% of residual variances that were not explained by the M2 model. The prediction accuracies from the CV1 scheme were smaller than both the CV2 and CV0 schemes, which suggests a less likely scenario in successfully implementing large-scale GS using newly developed lines that have not yet been phenotyped at any of the environments. The moderate to very high accuracies obtained with the CV2 and CV0 schemes, however, demonstrated highly likely scenarios for successfully implementing GS to develop disease-resistant spring wheat germplasm either by reducing the number of lines evaluated in each environment or predicting the performance of lines in future environments using data from some other environments. As far as we are aware, this is the first comprehensive genomic prediction study that has assessed the predictive ability of the reaction norm models across all five major wheat diseases in spring wheat and common bunt in wheat for the first time, which provides highly valuable information to breeders.

## Figures and Tables

**Table 1:** Descriptive statistics based on best linear unbiased estimators (BLUEs) and broad-sense heritability of three spring wheat populations evaluated for reactions to diseases in field conditions.

Population ^1^	Trait	No. of Environments	Correlation ^2^		Individual Environments ^3^			All Environments ^3^			Broad-Sense Heritability
			Range	Mean	Range	Mean	Std	Range	Mean	Std	
BVC	Stripe rust	8	0.10–0.81	0.52	1.0–9.0	3.7	2.5	1.2–8.1	3.76	1.80	0.89
	Leaf rust	4	0.43–0.75	0.55	1.0–9.0	3.6	2.3	1.0–8.1	3.52	1.78	0.78
	Leaf spot	3	0.07–0.28	0.09	1.0–9.0	4.1	2.0	2.2–6.8	4.12	0.92	0.22
	Common bunt	2	0.50–0.64	0.57	1.0–9.0	2.5	1.8	1.0–7.9	2.48	1.61	0.69
	FHB incidence	6	0.12–0.56	0.28	1.0–9.0	5.9	2.8	2.4–7.5	5.81	0.74	0.29
	FHB severity	6	0.11–0.67	0.41	1.0–9.0	3.8	2.1	1.8–6.8	4.15	0.94	0.58
	FHB index	6	0.10–0.68	0.42	0.5–82.1	26.3	20.4	3.7–49.6	25.97	9.11	0.50
PAC	Stripe rust	8	0.14–0.66	0.42	1.0–9.0	2.1	1.4	1.1–5.7	2.07	0.79	0.72
	Leaf rust	3	0.45–0.99	0.64	1.0–6.9	1.7	1.1	1.0–5.8	1.74	0.94	0.82
	Leaf spot	4	0.52–1.00	0.66	1.4–8.3	4.4	1.3	2.3–7.1	4.45	1.10	0.85
	Common bunt	3	0.76–1.00	0.84	1.0–7.5	1.6	1.1	1.0–6.0	1.59	1.06	0.90
ACG	Stripe rust	3	0.38–0.50	0.46	1.0–9.0	3.9	2.0	1.5–7.9	3.85	1.33	0.62
	Leaf rust	3	0.18–0.29	0.24	1.0–8.5	4.2	1.6	1.9–6.1	3.44	0.82	0.44
	Leaf spot	3	0.10–0.44	0.24	1.0–9.0	6.2	1.7	3.0–8.7	6.00	1.30	0.57
	Common bunt	3	0.22–0.37	0.32	1.0–7.0	2.3	1.4	1.0–4.9	1.79	0.82	0.52

^1^ BVC: association mapping panel; PAC and ACG: recombinant inbred lines derived from a cross between Peace × Carberry, and Attila × CDC Go, respectively. ^2^ Within-trait phenotypic correlation coefficients between pairs of environments (site × year combinations). ^3^ Range: minimum and maximum scores; Std: standard deviation of the mean.

**Table 2:** Mean prediction accuracies obtained using three random cross-validation schemes (CV1, CV2, and CV0) and three models (M1, M2, and M3). See Table S5 for details of prediction accuracies.

		CV1						CV2						CV0					
		M1		M2		M3		M1		M2		M3		M1		M2		M3	
Population ^1^	Trait ^2^	Mean	Std ^3^	Mean	Std	Mean	Std	Mean	Std	Mean	Std	Mean	Std	Mean	Std	Mean	Std	Mean	Std
BVC	Bunt	−0.11	0.07	0.43	0.02	0.44	0.03	0.56	0.03	0.62	0.02	0.62	0.03	0.62	0.02	0.63	0.00	0.62	0.04
	FHB inc.	−0.13	0.06	0.22	0.09	0.27	0.13	0.41	0.12	0.43	0.13	0.45	0.13	0.44	0.14	0.45	0.14	0.45	0.14
	FHB index	−0.10	0.07	0.39	0.16	0.40	0.18	0.56	0.13	0.58	0.15	0.63	0.16	0.59	0.16	0.59	0.17	0.60	0.16
	FHB sev.	−0.11	0.07	0.42	0.14	0.41	0.17	0.58	0.15	0.59	0.16	0.62	0.18	0.60	0.17	0.61	0.18	0.61	0.18
	Lr	−0.09	0.08	0.49	0.11	0.48	0.12	0.58	0.09	0.64	0.10	0.64	0.09	0.63	0.13	0.65	0.12	0.59	0.05
	Ls	−0.13	0.05	0.18	0.20	0.38	0.16	0.07	0.10	0.21	0.18	0.41	0.15	0.12	0.10	0.11	0.14	0.14	0.11
	Yr	−0.10	0.07	0.48	0.08	0.49	0.09	0.75	0.07	0.76	0.07	0.76	0.06	0.77	0.08	0.76	0.07	0.77	0.08
PAC	Bunt	−0.10	0.10	0.31	0.06	0.29	0.07	0.84	0.07	0.84	0.06	0.84	0.06	0.87	0.09	0.87	0.09	0.87	0.09
	Lr	−0.06	0.07	0.31	0.08	0.29	0.11	0.70	0.19	0.71	0.20	0.70	0.20	0.77	0.27	0.73	0.24	0.77	0.27
	Ls	−0.03	0.08	0.33	0.04	0.32	0.07	0.73	0.12	0.73	0.11	0.74	0.10	0.75	0.14	0.74	0.13	0.75	0.13
	Yr	−0.09	0.07	0.26	0.07	0.26	0.10	0.54	0.09	0.54	0.09	0.55	0.08	0.56	0.10	0.56	0.08	0.56	0.10
ACG	Bunt	−0.14	0.07	0.02	0.07	0.02	0.09	0.39	0.05	0.39	0.06	0.34	0.08	0.41	0.04	0.42	0.06	0.42	0.05
	Lr	−0.14	0.07	0.13	0.05	0.11	0.06	0.27	0.04	0.29	0.03	0.25	0.04	0.30	0.03	0.33	0.01	0.32	0.02
	Ls	−0.16	0.06	0.04	0.10	0.08	0.07	0.28	0.12	0.28	0.13	0.31	0.08	0.32	0.14	0.30	0.16	0.32	0.15
	Yr	−0.12	0.09	0.25	0.06	0.29	0.08	0.53	0.05	0.54	0.05	0.56	0.06	0.54	0.06	0.53	0.05	0.54	0.05

^1^ BVC: association mapping panel; PAC and ACG: recombinant inbred lines derived from a cross between Peace × Carberry, and Attila × CDC Go, respectively. ^2^ Yr: yellow (stripe) rust; Lr: leaf rust; Ls: leaf spot; FHB: common bunt; FHB inc.: Fusarium head blight (FHB) incidence; FHB sev.: FHB severity. ^3^ Std: standard deviation of the mean. M1, M3, and M3 represent the baseline model, the main effect reaction norm model, and the model that incorporated GE interactions, respectively.

## Data Availability

All relevant results are included within this article and its files. The original phenotype and genotype data are available on request from the corresponding author. The data are not publicly available due to (a) the use of several varieties from the private sector with restricted breeder’s rights; and (b) other manuscripts that are under review and preparation.

## Supplementary Materials

The following supporting information can be downloaded at: https://www.mdpi.com/article/10.3390/genes13040565/s1, Figure S1: Scatter plots of best linear unbiased estimators of Fusarium head blight (FHB) incidence, severity, and index based on six environments; Figure S2: Scatter plots of the best linear unbiased estimators of Fusarium head blight (FHB) incidence, severity, and index based on (a) six environments, and (b) individual environments; Figure S3: Frequency distribution of the best linear unbiased estimators (BLUEs) of disease scores in three spring wheat populations evaluated under field conditions; Figure S4: A plot of PC1 and PC2 to show the within-population structure of (a) an association mapping panel (BVC) and two recombinant inbred line populations derived from Attila × CDC Go (ACG) and Peace × Carberry (PAC); (b) a plot of the different wheat classes within the BVC association mapping panel; Figure S5: Regression plots of prediction accuracies based on the best linear unbiased estimators computed from all environments; Figure S6: Comparison of prediction accuracies of three populations evaluated for disease reaction at multiple field environments using three models (M1, M2, and M3) and three random cross-validation schemes (CV0, CV1, and CV2); Table S1: (A) Summary of the two recombinant inbred line populations and association mapping panel used in the present study; (B) summary of the Canadian spring wheat association mapping panel; Table S2: Summary of polymorphic markers used in the present study; Table S3: Summary statistics and broad-sense heritability of seven disease-related traits in three spring wheat populations evaluated at 2–8 field environments; Table S4: Partitioning of variance components into environments (E), wheat lines (L), molecular markers or genomics (G), and interactions between markers and environments (G × E), and residual (unexplained) variance using three reaction norm models (M1, M2, and M3 models). Values are in percentages; Table S5: (A) Prediction accuracies of five wheat diseases recorded in three spring wheat populations in 2–8 field environments. The results were obtained for individual environments using three cross-validation schemes (CV0, CV1, and CV2), three models (M1, M2, and M3), and ten iterations. Populations are as follows—BVC: association mapping panel; ACG: Attila × CDC Go; PAC: Peace × Carberry. (B) Average prediction accuracies per traits and individual environment based on three cross-validation schemes (CV0, CV1, and CV2), and three models (M1, M2, and M3). (C) Average prediction accuracies per trait regardless of populations and environments based on three cross-validation schemes (CV0, CV1, and CV2) and three models (M1, M2, and M3). (D) Analysis of variance based on the general linear model (GLM) to compare prediction accuracies obtained for all three populations and seven traits summarized in Table S5A.
