# Supplementary material for: Genomic Predictions for Common Bunt, FHB, Stripe Rust, Leaf Rust, and Leaf Spotting Resistance in Spring Wheat
Source: Genes (Basel). 2022 Mar 23;13(4):565. doi: 10.3390/genes13040565 (PMC9032109; doi:10.3390/genes13040565)
Supplement: Supplementary file 1 [file genes-13-00565-s001.zip › Supplementary Figures S1-S6 R1.pptx]

## Slide 1
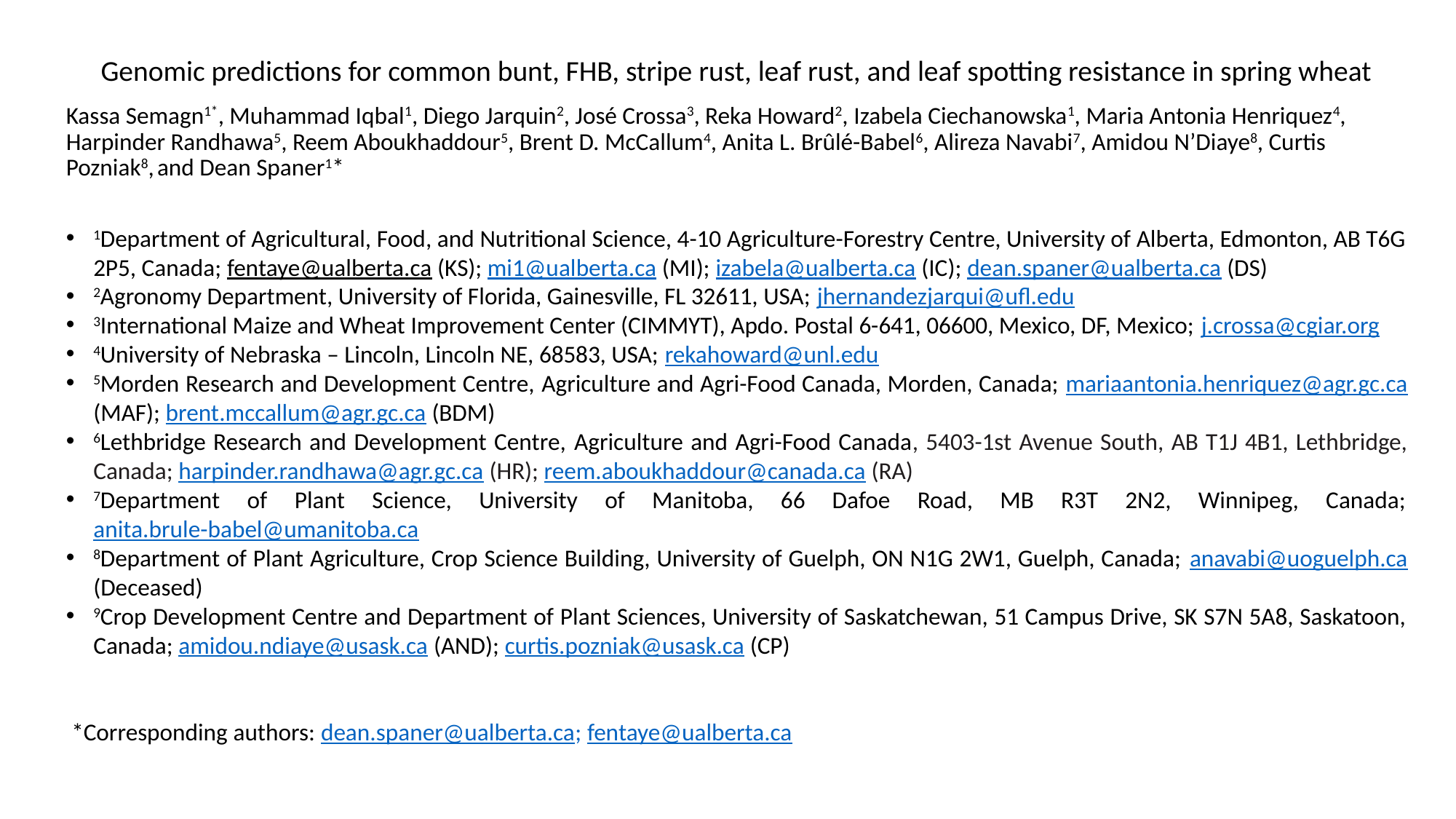

Genomic predictions for common bunt, FHB, stripe rust, leaf rust, and leaf spotting resistance in spring wheat
Kassa Semagn1*, Muhammad Iqbal1, Diego Jarquin2, José Crossa3, Reka Howard2, Izabela Ciechanowska1, Maria Antonia Henriquez4, Harpinder Randhawa5, Reem Aboukhaddour5, Brent D. McCallum4, Anita L. Brûlé-Babel6, Alireza Navabi7, Amidou N’Diaye8, Curtis Pozniak8, and Dean Spaner1*
1Department of Agricultural, Food, and Nutritional Science, 4-10 Agriculture-Forestry Centre, University of Alberta, Edmonton, AB T6G 2P5, Canada; fentaye@ualberta.ca (KS); mi1@ualberta.ca (MI); izabela@ualberta.ca (IC); dean.spaner@ualberta.ca (DS)
2Agronomy Department, University of Florida, Gainesville, FL 32611, USA; jhernandezjarqui@ufl.edu
3International Maize and Wheat Improvement Center (CIMMYT), Apdo. Postal 6-641, 06600, Mexico, DF, Mexico; j.crossa@cgiar.org
4University of Nebraska – Lincoln, Lincoln NE, 68583, USA; rekahoward@unl.edu
5Morden Research and Development Centre, Agriculture and Agri-Food Canada, Morden, Canada; mariaantonia.henriquez@agr.gc.ca (MAF); brent.mccallum@agr.gc.ca (BDM)
6Lethbridge Research and Development Centre, Agriculture and Agri-Food Canada, 5403-1st Avenue South, AB T1J 4B1, Lethbridge, Canada; harpinder.randhawa@agr.gc.ca (HR); reem.aboukhaddour@canada.ca (RA)
7Department of Plant Science, University of Manitoba, 66 Dafoe Road, MB R3T 2N2, Winnipeg, Canada; anita.brule-babel@umanitoba.ca
8Department of Plant Agriculture, Crop Science Building, University of Guelph, ON N1G 2W1, Guelph, Canada; anavabi@uoguelph.ca (Deceased)
9Crop Development Centre and Department of Plant Sciences, University of Saskatchewan, 51 Campus Drive, SK S7N 5A8, Saskatoon, Canada; amidou.ndiaye@usask.ca (AND); curtis.pozniak@usask.ca (CP)
 *Corresponding authors: dean.spaner@ualberta.ca; fentaye@ualberta.ca

## Slide 2
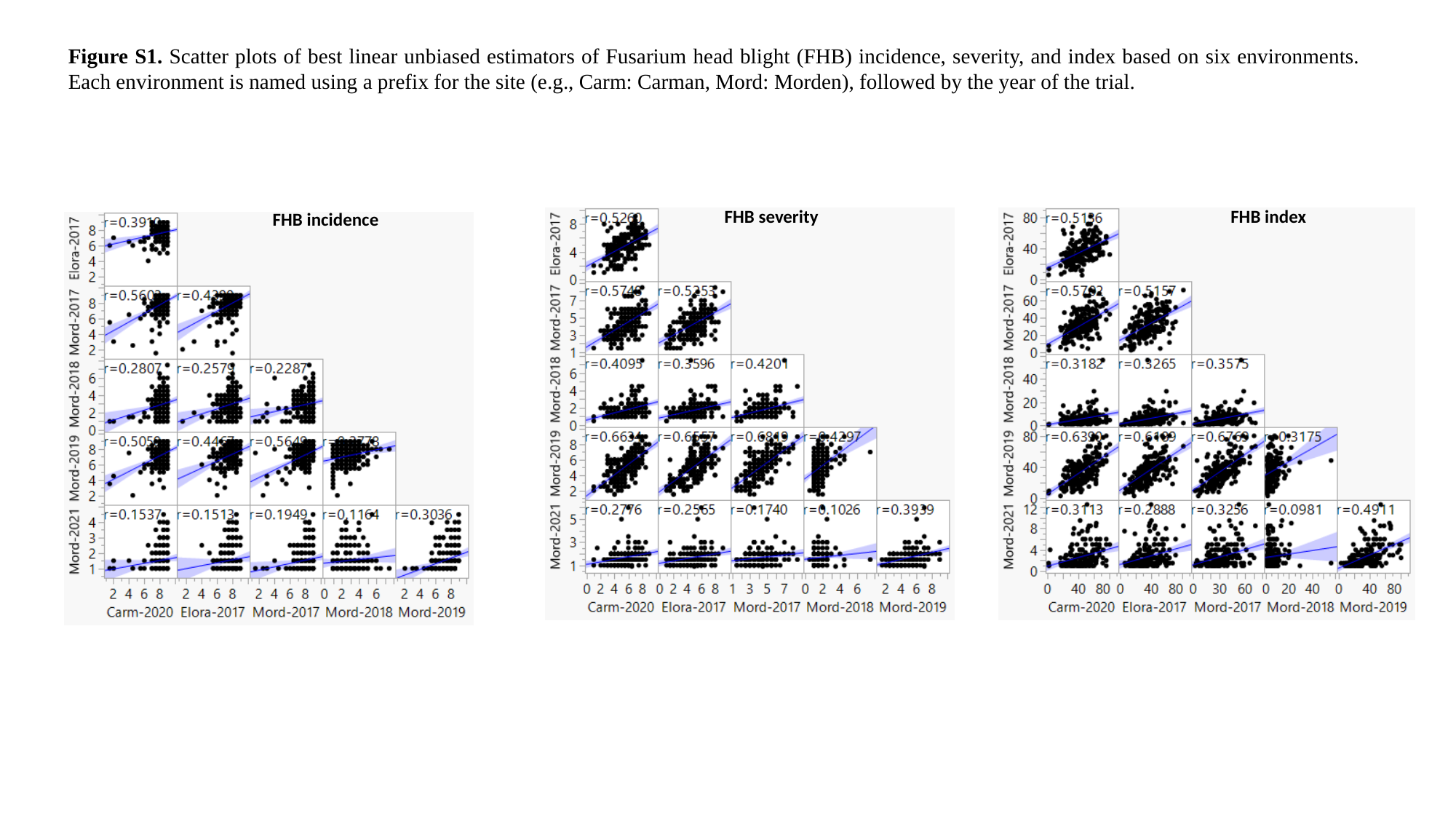

Figure S1. Scatter plots of best linear unbiased estimators of Fusarium head blight (FHB) incidence, severity, and index based on six environments. Each environment is named using a prefix for the site (e.g., Carm: Carman, Mord: Morden), followed by the year of the trial.
FHB severity
FHB index
FHB incidence

## Slide 3
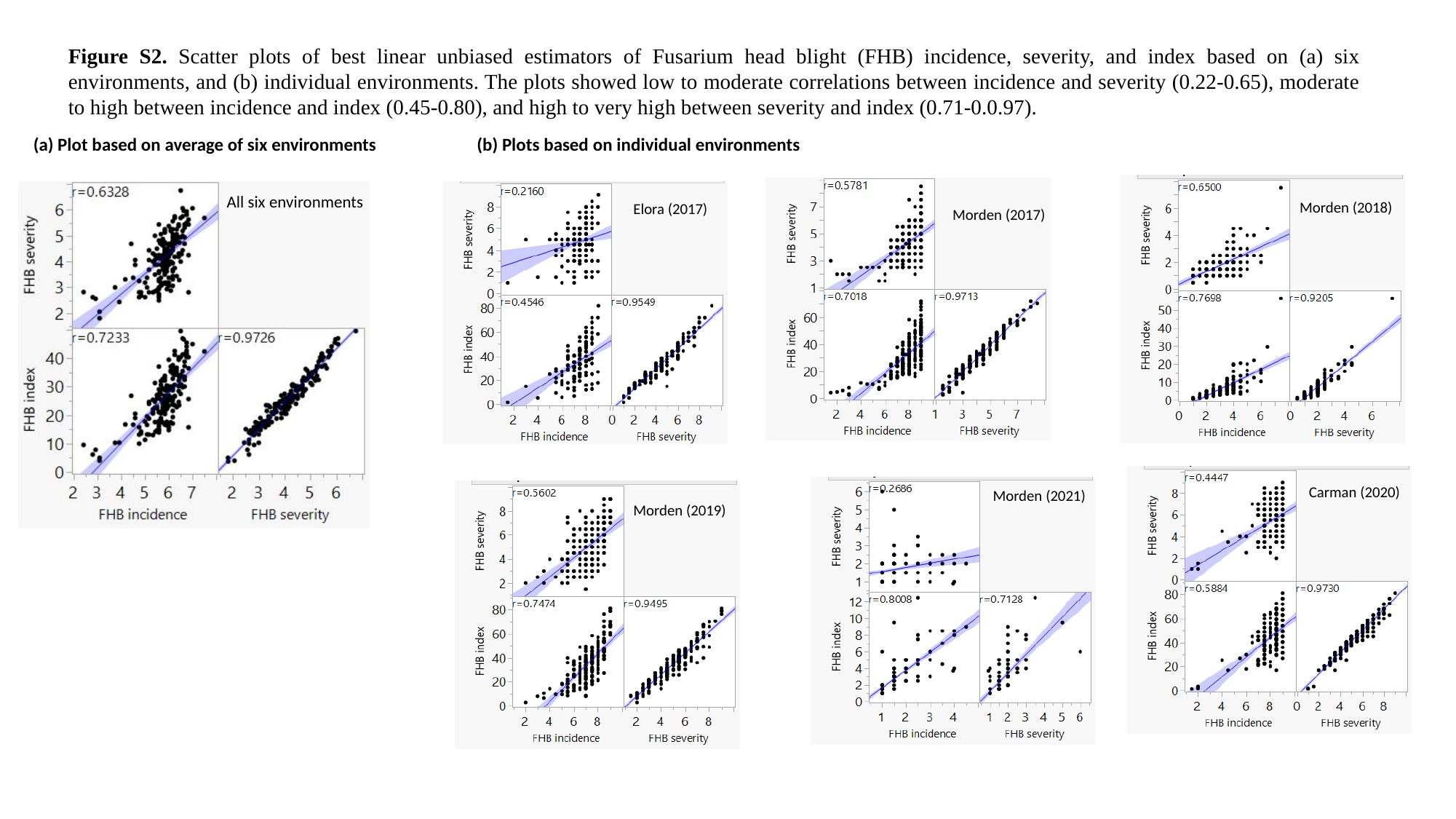

Figure S2. Scatter plots of best linear unbiased estimators of Fusarium head blight (FHB) incidence, severity, and index based on (a) six environments, and (b) individual environments. The plots showed low to moderate correlations between incidence and severity (0.22-0.65), moderate to high between incidence and index (0.45-0.80), and high to very high between severity and index (0.71-0.0.97).
(a) Plot based on average of six environments
(b) Plots based on individual environments
Morden (2018)
Morden (2017)
Elora (2017)
All six environments
Carman (2020)
Morden (2021)
Morden (2019)

## Slide 4
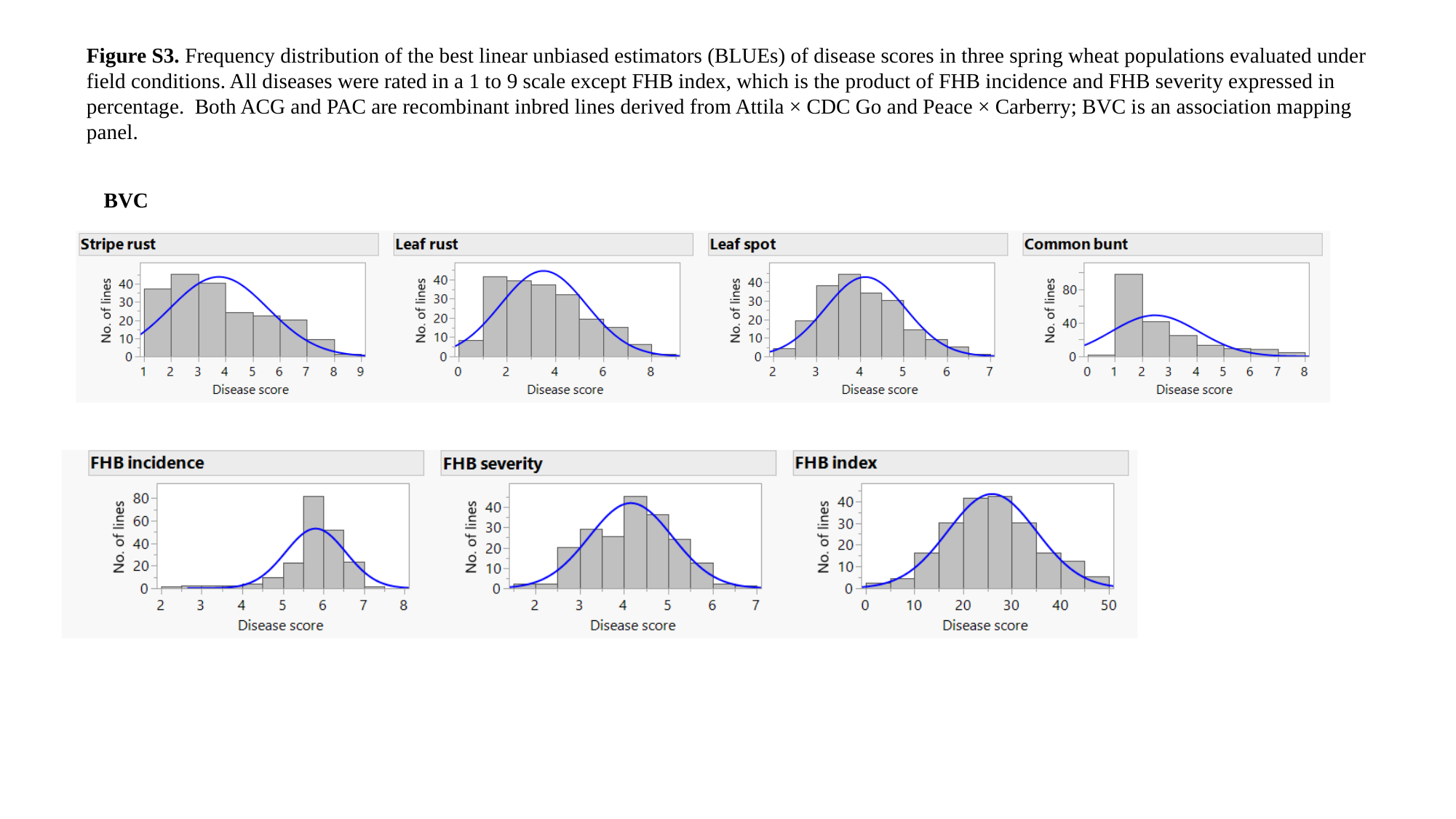

Figure S3. Frequency distribution of the best linear unbiased estimators (BLUEs) of disease scores in three spring wheat populations evaluated under field conditions. All diseases were rated in a 1 to 9 scale except FHB index, which is the product of FHB incidence and FHB severity expressed in percentage. Both ACG and PAC are recombinant inbred lines derived from Attila × CDC Go and Peace × Carberry; BVC is an association mapping panel.
BVC

## Slide 5
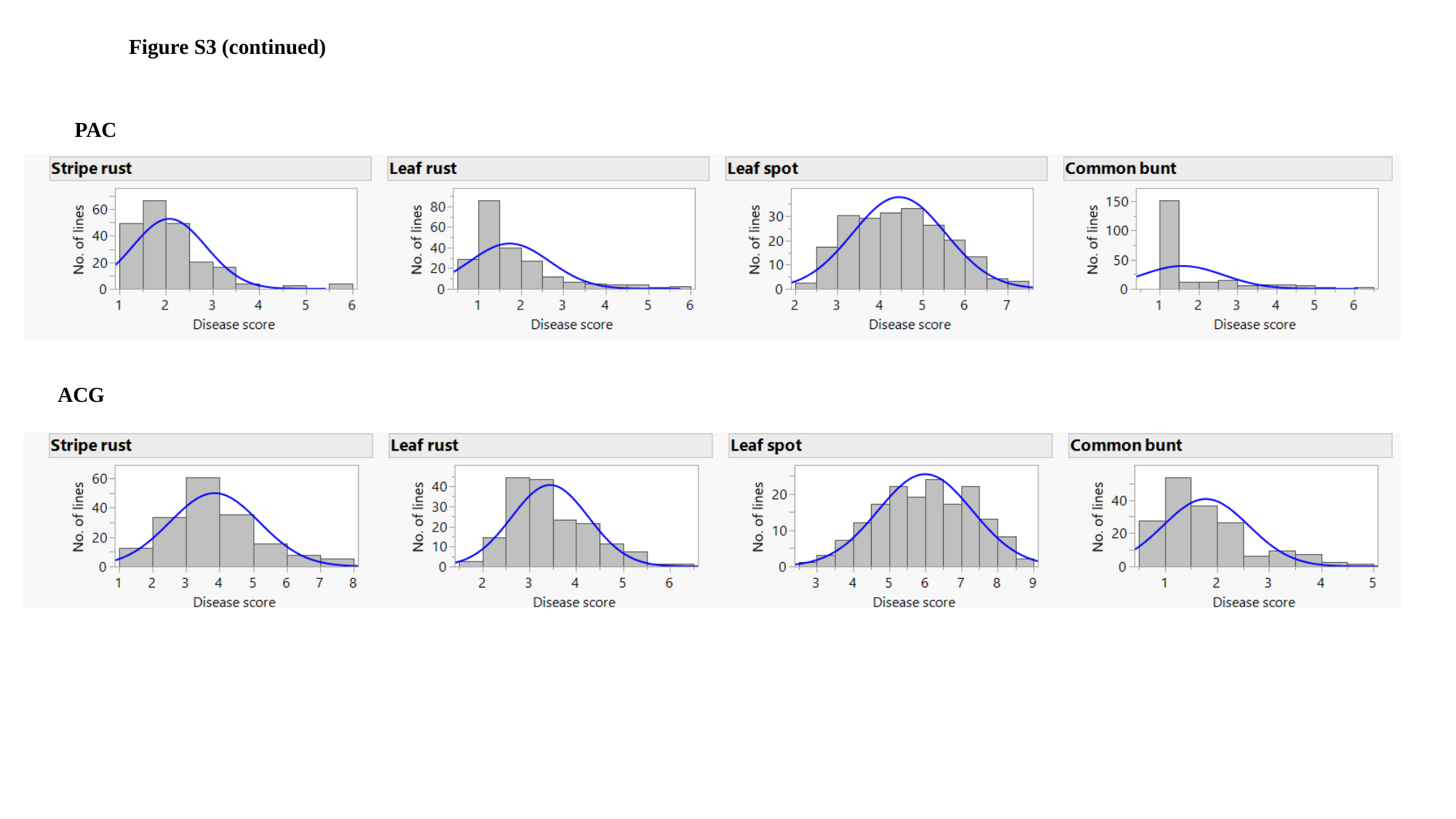

Figure S3 (continued)
PAC
ACG

## Slide 6
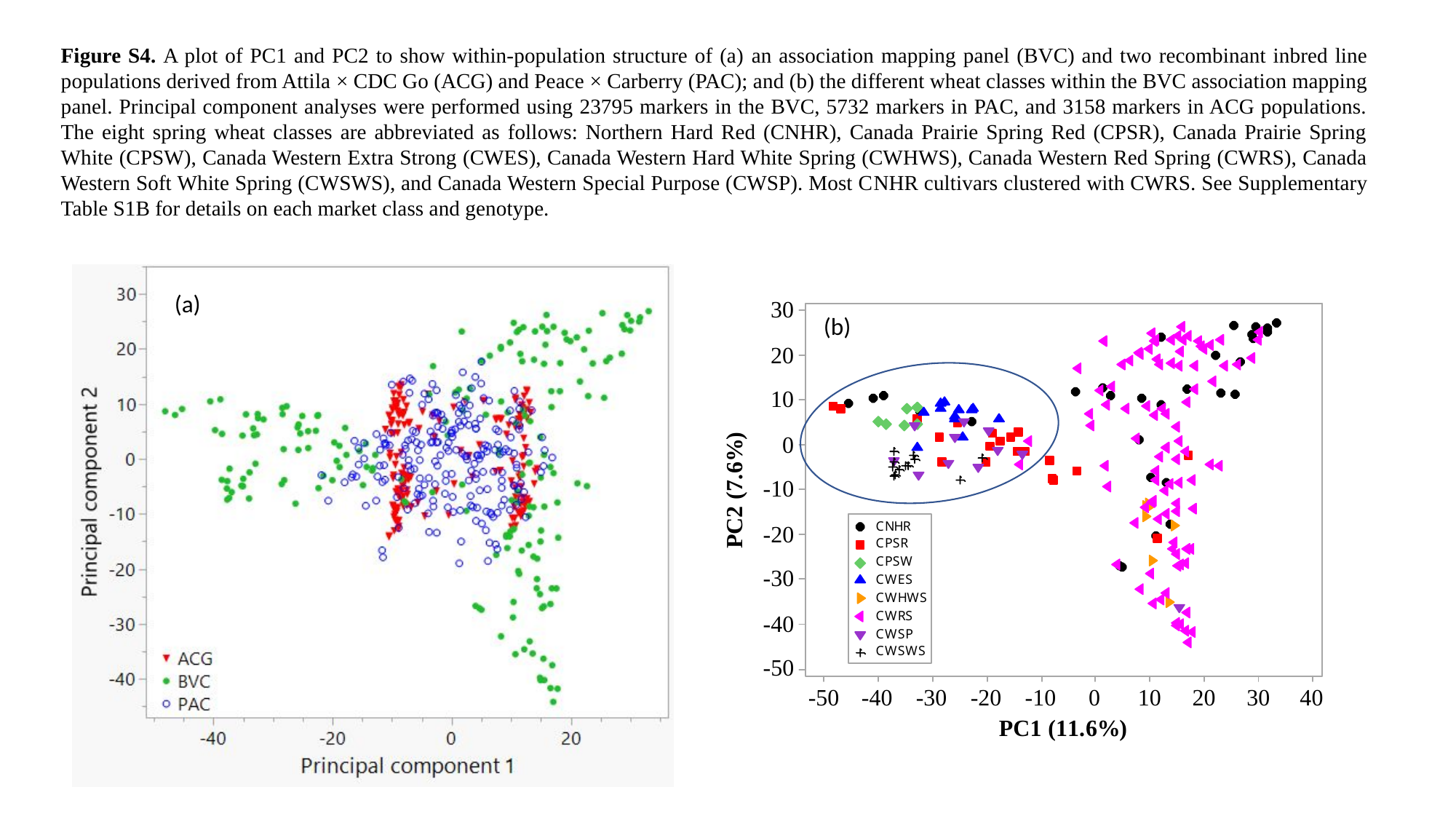

Figure S4. A plot of PC1 and PC2 to show within-population structure of (a) an association mapping panel (BVC) and two recombinant inbred line populations derived from Attila × CDC Go (ACG) and Peace × Carberry (PAC); and (b) the different wheat classes within the BVC association mapping panel. Principal component analyses were performed using 23795 markers in the BVC, 5732 markers in PAC, and 3158 markers in ACG populations. The eight spring wheat classes are abbreviated as follows: Northern Hard Red (CNHR), Canada Prairie Spring Red (CPSR), Canada Prairie Spring White (CPSW), Canada Western Extra Strong (CWES), Canada Western Hard White Spring (CWHWS), Canada Western Red Spring (CWRS), Canada Western Soft White Spring (CWSWS), and Canada Western Special Purpose (CWSP). Most CNHR cultivars clustered with CWRS. See Supplementary Table S1B for details on each market class and genotype.
(a)
(b)

## Slide 7
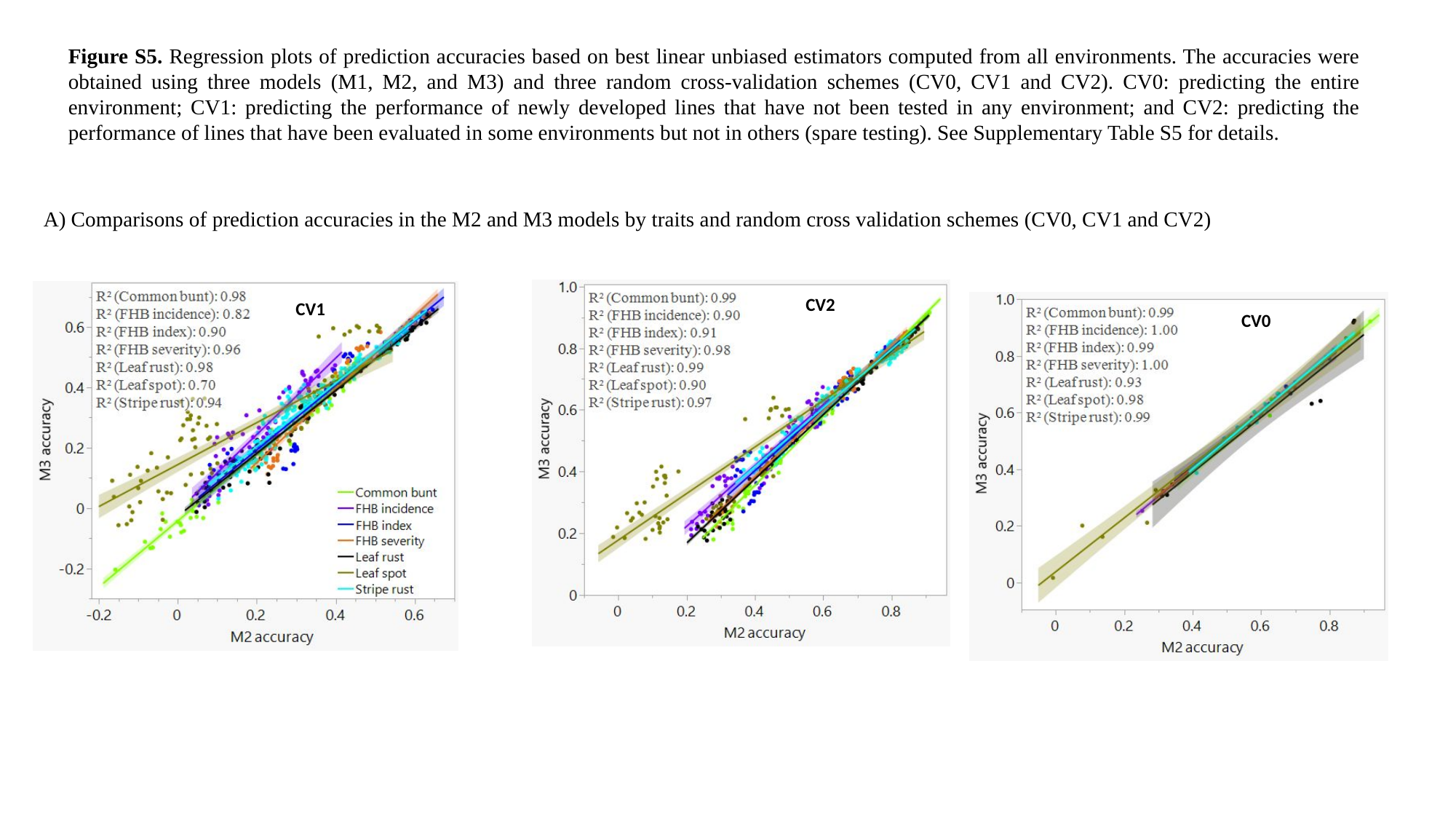

Figure S5. Regression plots of prediction accuracies based on best linear unbiased estimators computed from all environments. The accuracies were obtained using three models (M1, M2, and M3) and three random cross-validation schemes (CV0, CV1 and CV2). CV0: predicting the entire environment; CV1: predicting the performance of newly developed lines that have not been tested in any environment; and CV2: predicting the performance of lines that have been evaluated in some environments but not in others (spare testing). See Supplementary Table S5 for details.
A) Comparisons of prediction accuracies in the M2 and M3 models by traits and random cross validation schemes (CV0, CV1 and CV2)
CV2
CV1
CV0

## Slide 8
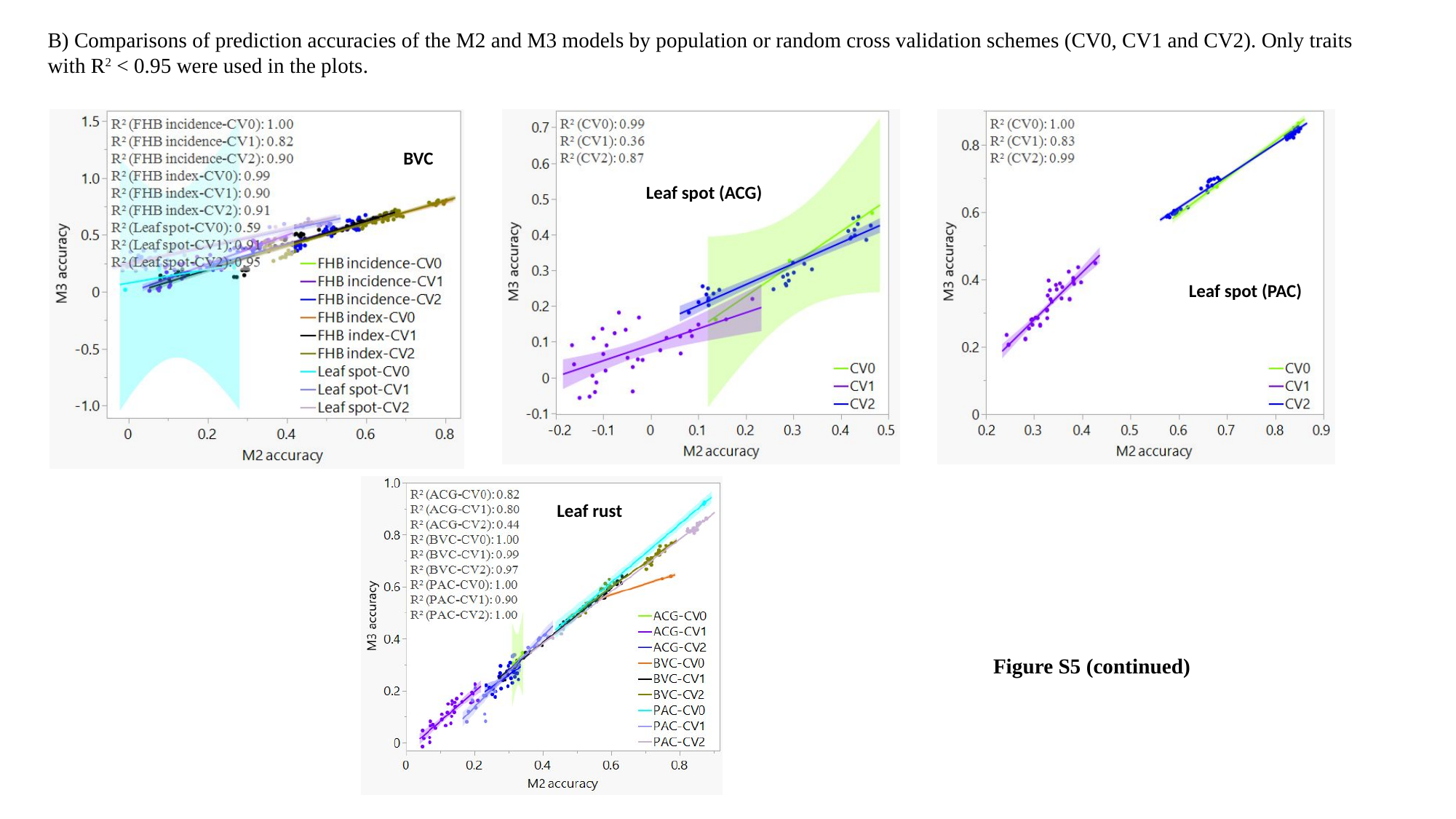

B) Comparisons of prediction accuracies of the M2 and M3 models by population or random cross validation schemes (CV0, CV1 and CV2). Only traits with R2 < 0.95 were used in the plots.
Leaf spot (ACG)
Leaf spot (PAC)
BVC
Leaf rust
Figure S5 (continued)

## Slide 9
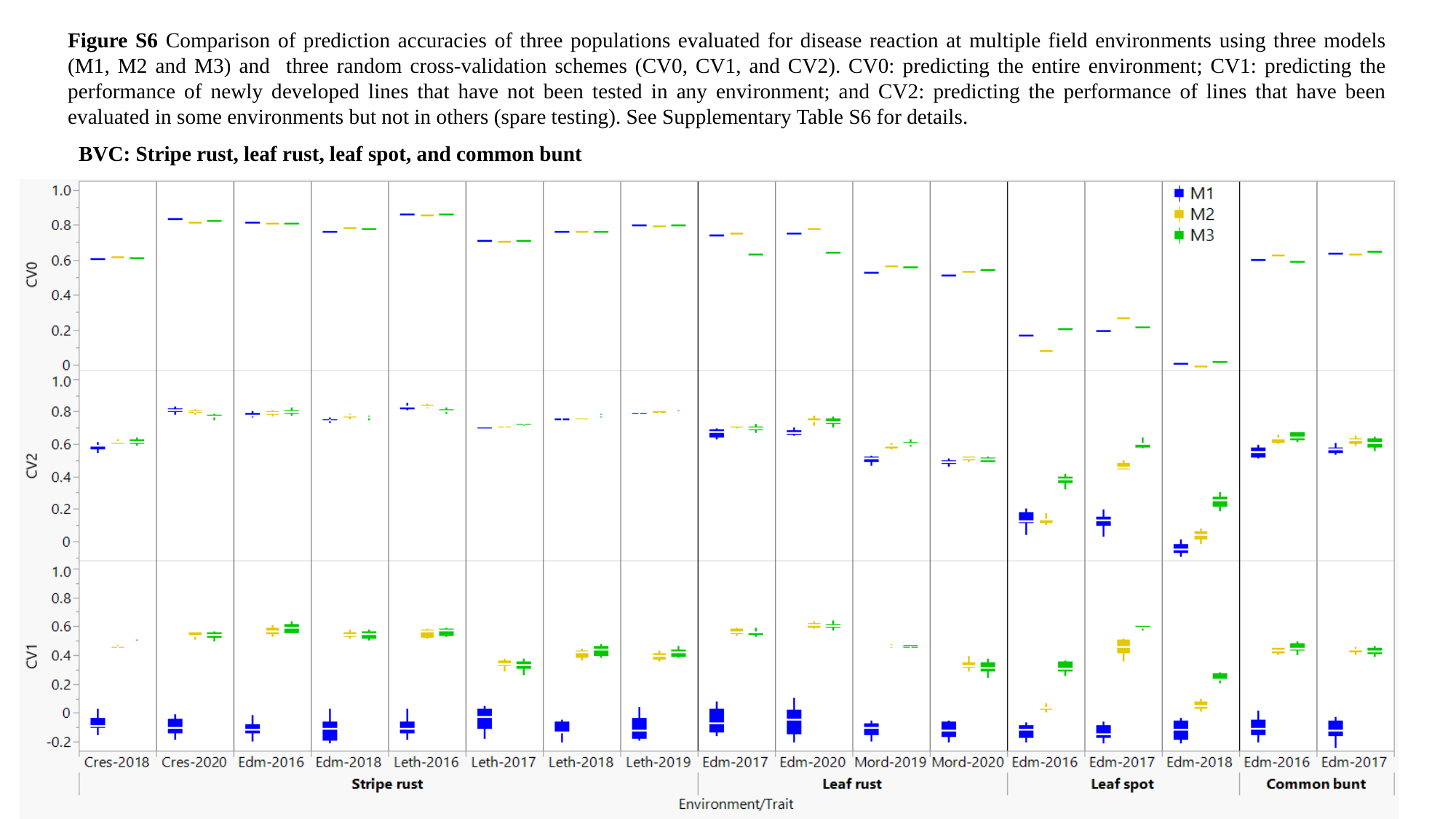

Figure S6 Comparison of prediction accuracies of three populations evaluated for disease reaction at multiple field environments using three models (M1, M2 and M3) and three random cross-validation schemes (CV0, CV1, and CV2). CV0: predicting the entire environment; CV1: predicting the performance of newly developed lines that have not been tested in any environment; and CV2: predicting the performance of lines that have been evaluated in some environments but not in others (spare testing). See Supplementary Table S6 for details.
BVC: Stripe rust, leaf rust, leaf spot, and common bunt

## Slide 10
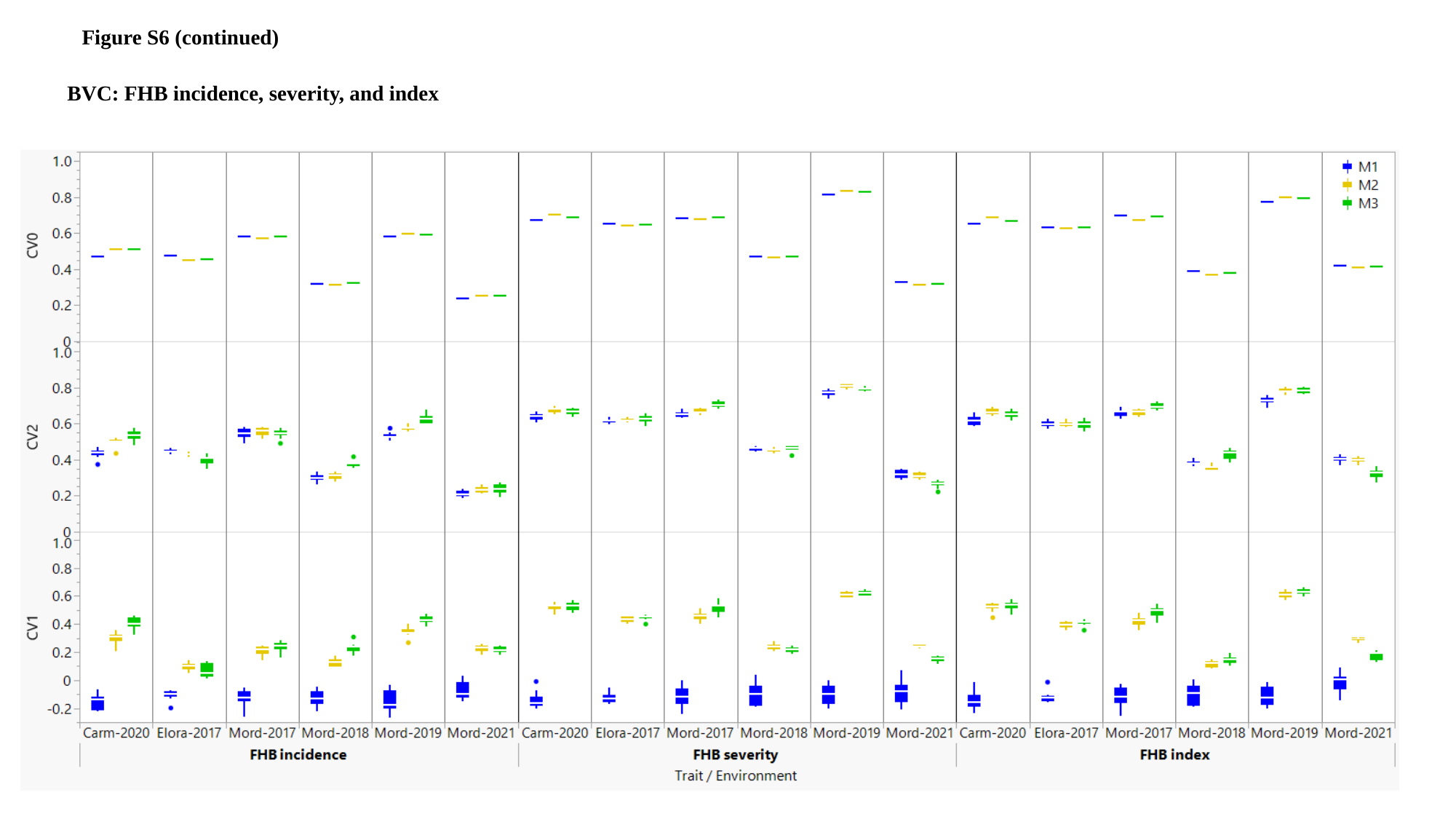

Figure S6 (continued)
BVC: FHB incidence, severity, and index

## Slide 11
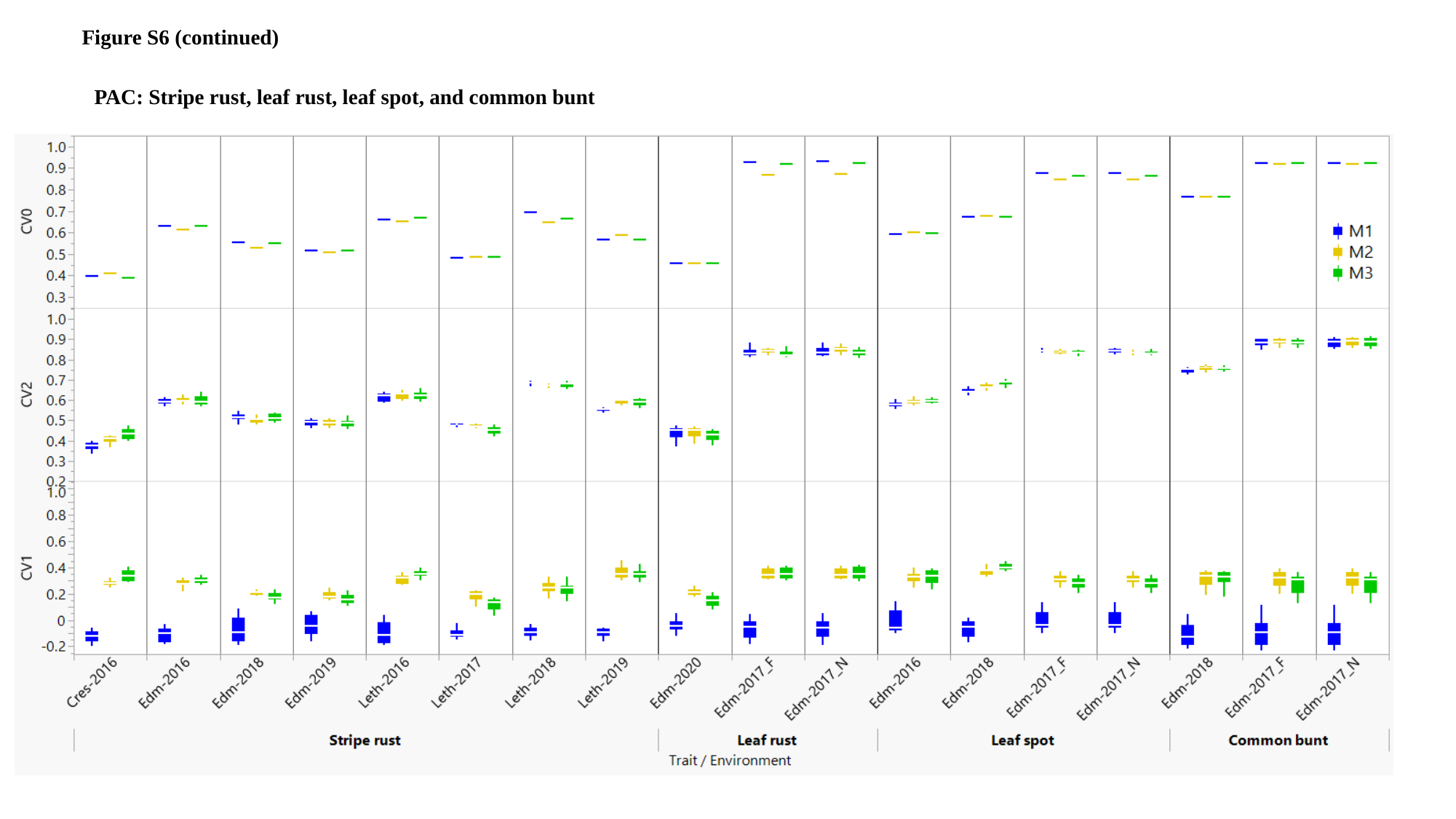

Figure S6 (continued)
PAC: Stripe rust, leaf rust, leaf spot, and common bunt

## Slide 12
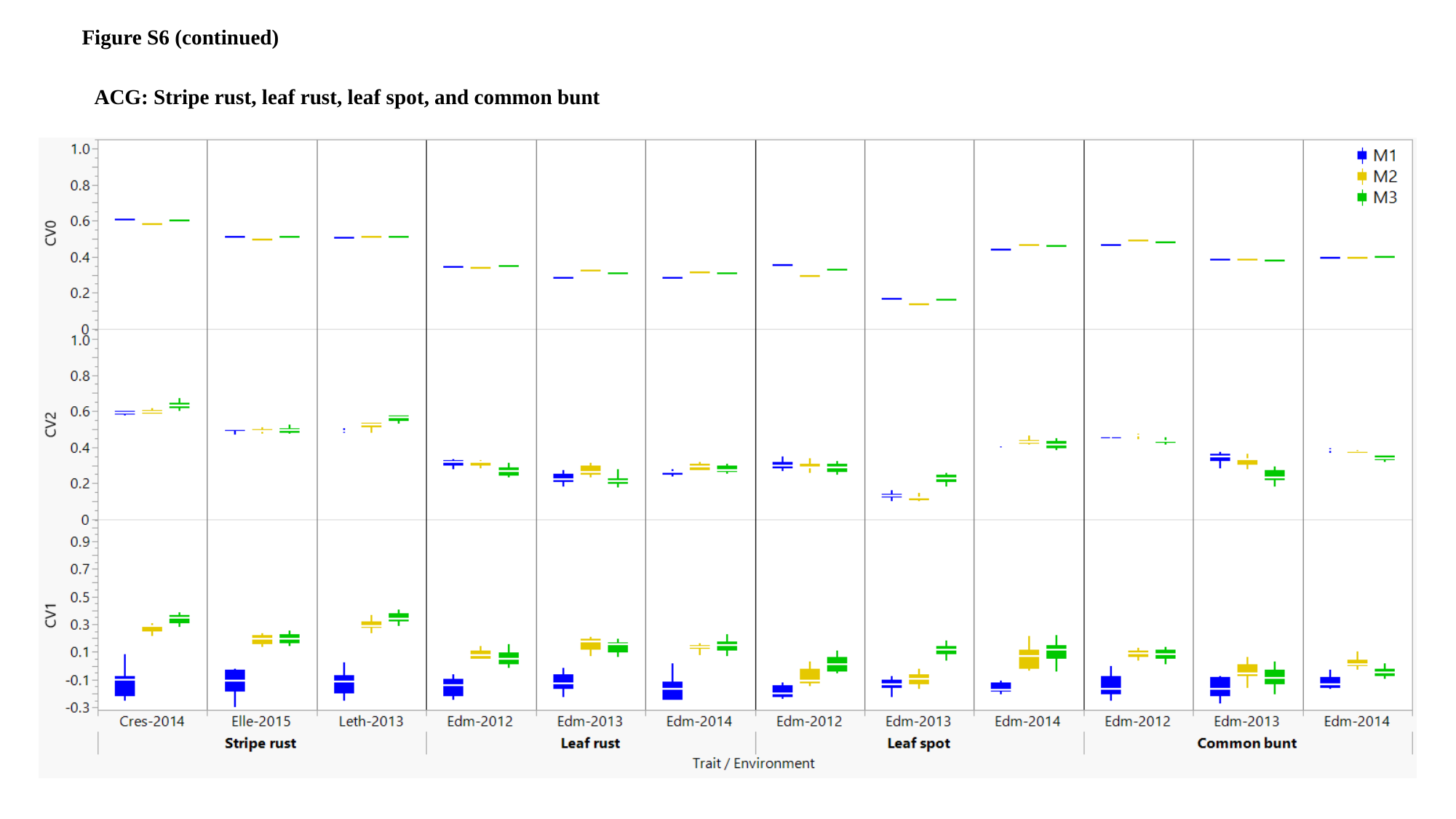

Figure S6 (continued)
ACG: Stripe rust, leaf rust, leaf spot, and common bunt
